# Bacterial communities in temperate and polar coastal sands are seasonally stable

**DOI:** 10.1038/s43705-021-00028-w

**Published:** 2021-06-28

**Authors:** Sebastian Miksch, Mirja Meiners, Anke Meyerdierks, David Probandt, Gunter Wegener, Jürgen Titschack, Maria A. Jensen, Andreas Ellrott, Rudolf Amann, Katrin Knittel

**Affiliations:** 1 Max Planck Institute for Marine Microbiology, Bremen, Germany; 2 MARUM, Center for Marine Environmental Sciences, University of Bremen, Bremen, Germany; 3 Alfred Wegener Institute, Helmholtz Centre for Polar and Marine Research, Bremerhaven, Germany; 4 Senckenberg am Meer, Wilhelmshaven, Germany; 5 UNIS, The University Centre in Svalbard, Longyearbyen, Norway

**Keywords:** Microbial ecology, Molecular ecology

## Abstract

Coastal sands are biocatalytic filters for dissolved and particulate organic matter of marine and terrestrial origin, thus, acting as centers of organic matter transformation. At high temporal resolution, we accessed the variability of benthic bacterial communities over two annual cycles at Helgoland (North Sea), and compared it with seasonality of communities in Isfjorden (Svalbard, 78°N) sediments, where primary production does not occur during winter. Benthic community structure remained stable in both, temperate and polar sediments on the level of cell counts and 16S rRNA-based taxonomy. Actinobacteriota of uncultured Actinomarinales and Microtrichales were a major group, with 8 ± 1% of total reads (Helgoland) and 31 ± 6% (Svalbard). Their high activity (frequency of dividing cells 28%) and in situ cell numbers of >10% of total microbes in Svalbard sediments, suggest Actinomarinales and Microtrichales as key heterotrophs for carbon mineralization. Even though Helgoland and Svalbard sampling sites showed no phytodetritus-driven changes of the benthic bacterial community structure, they harbored significantly different communities (*p* < 0.0001, *r* = 0.963). The temporal stability of benthic bacterial communities is in stark contrast to the dynamic succession typical of coastal waters, suggesting that pelagic and benthic bacterial communities respond to phytoplankton productivity very differently.

## Introduction

Sandy sediments cover approximately 70% of continental shelves [1]. These sediments are characterized by high permeability and advective transport by which bottom water is pumped through the pore space [2]. Thus, they work as expansive filter systems: suspended particles, algae, and bacteria are transported with the penetrating water into the sediment, where they become trapped in the pores [1, 3–5]. Simultaneously, electron acceptors and nutrients are provided to benthic microbial communities, strongly enhancing bentho-pelagic coupling [6]. Continental shelf areas are highly productive ecosystems, contributing 15–21% to global primary production [7]. In shallow areas, up to 50% of the pelagic primary production can reach the seafloor [8, 9]. Active heterotrophic bacteria rapidly mineralize the settled phytodetritus as well as fresh organic matter derived from benthic primary production [1, 2, 10], as demonstrated by high extracellular enzyme activities, bacterial carbon production, and organic matter mineralization [11–13].

Despite the close linkage of water column and sediments, major compositional differences in microbial communities are evident [14–16]. For example, SAR11, SAR86, and “*Candidatus* Actinomarina” are abundant members of bacterioplankton, but rare in sediments. Based on an analysis of 509 samples spanning the global surface oceans to the deep-sea floor, pelagic, and benthic communities share only 10% of bacterial types defined at 3% sequence similarity level [16]. Compositional differences between pelagic and benthic microbial communities are accompanied by differences in function [15, 17]. Patterns of enzyme activities differed and showed a more diversified enzyme spectrum in sediments.

In the water column, phytoplankton blooms in spring and summer induce changes in bacterioplankton community structure [18–20]. For example, in the southern North Sea, relative cell numbers of *Polaribacter, Ulvibacter*, and SAR92 increased by factors of 5–20, shortly after bloom events [19]. These community dynamics were recurrent, indicating a phytodetritus-driven seasonality [19–21]. Knowledge of seasonality of benthic microbes, in contrast, is sparse. Seasonal changes in organic matter mineralization, extracellular enzyme activities, and bacterial biomass production rates were mostly related to temperature and substrate availability in sediments [5, 11, 22–24]. Different substrate additions showed specific effects on benthic microbial communities in incubations with Arctic deep-sea sediments Hausgarten, 78.5–80°N [25]. Addition of chitin resulted in an increased activity of the community without major compositional change, whereas addition of phytodetritus caused a strong change of community composition. Overall, there are only few studies of seasonal changes of microbial diversity and in situ abundance of major taxa [13, 26–29], indicating a high turnover of rare organisms but an unchanged pattern of major taxa [26]. In surface sediments from English Channel, Tait and colleagues found indications for seasonality of Flavobacteriia [29]. However, all these studies were based only on 2–5 sampling dates or on a single year.

In order to assess at high temporal resolution the variability of sandy sediment bacterial communities over an annual cycle, we sampled a shallow coastal site at Helgoland Roads (North Sea, 54°N) 19 times over the course of two years. The sample intervals were 10–20 days apart, during the spring bloom, and 6–8 weeks for the rest of the year. Although phytoplankton at Helgoland exhibits a strong seasonality [30], metatranscriptomic analysis of sediments showed that 36–53% of mRNA transcripts were related to photosynthesis genes [31], indicating ongoing primary production either in the water column or in the sediments during winter. Therefore, in order to compare seasonality in sediments at a location where primary production does not occur during winter, we also visited Svalbard 6 times between December 2017 and September 2019. The Svalbard archipelago, at 76°–81°N, is sufficiently far North that primary productivity does not occur during the polar night [32].

We tested the hypothesis that phytodetrital input drives seasonal changes in benthic bacterial community structures of Svalbard and Helgoland sediments. In particular, we wanted to identify community changes typical of the strong seasonality found in higher latitudes. We expected changes to be more pronounced in Svalbard sediments than in Helgoland sediments. To quantitatively assess sedimentary communities, we initially established an automated microscopic system for enumeration of bacteria, and developed new probes for Actinobacteriota. The in situ abundances of major taxa were investigated by CARD-FISH and the bacterial diversity by 16S rRNA gene sequencing.

## Materials and methods

### Study sites and sampling

**Fig. 1:**
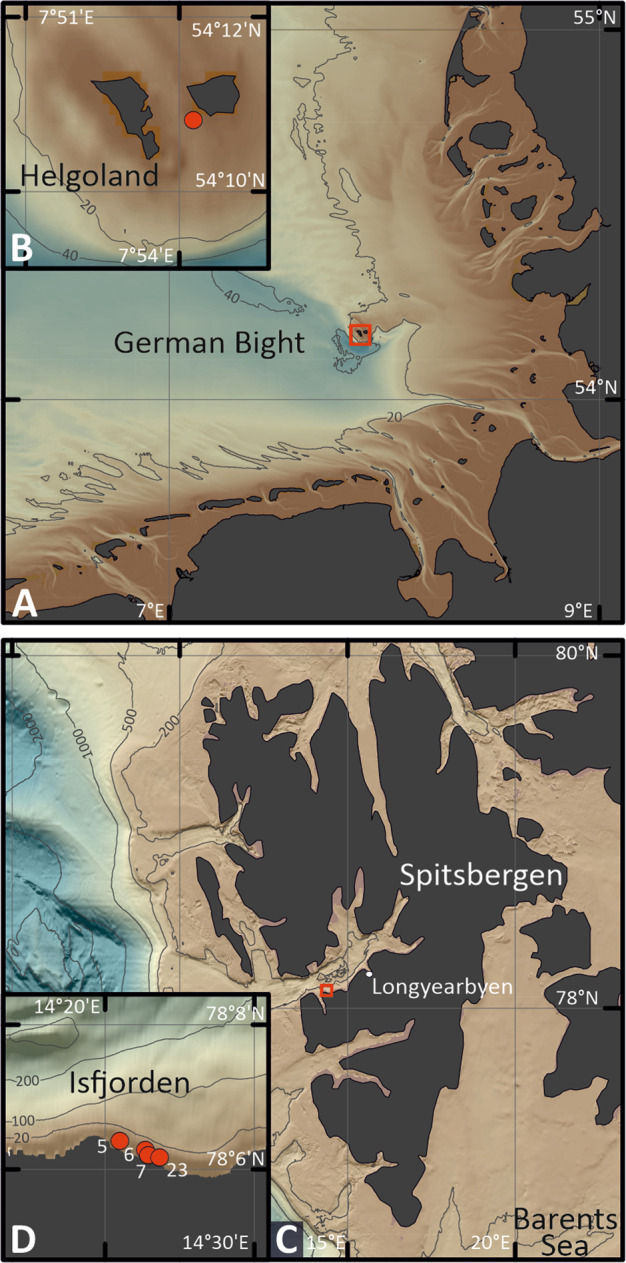
Map of sampling areas. **A**, **B** Helgoland Roads, North Sea (German Bight); **C**, **D** Isfjorden, an Arctic fjord in western Spitsbergen (archipelago of Svalbard, Arctic Ocean). Data sources: European Environment Agency, EEA coastline derived from EU-Hydro and GSHHG data; http://www.eea.europa.eu/data-and-maps/data/eea-coastline-for-analysis and EMODnet Bathymetry Consortium (2016, 2020): EMODnet Digital Bathymetry (DTM). 10.12770/bb6a87dd-e579-4036-abe1-e649cea9881a; 10.12770/c7b53704-999d-4721-b1a3-04ec60c87238.

At Helgoland, North Sea, (German Bight; 54.18°N, 7.90°E), sediment samples were obtained by scientific divers of the Alfred Wegner Institute (Bremerhaven, Germany) using push cores. The sampling site is located at Helgoland Roads between the main island and the dune (Fig. 1A). Water depth varied between ~3 and ~8 m. At Isfjorden, Svalbard, Arctic Ocean (Fig. 1B), sediments were obtained using a van Veen grab deployed from R/V *Farm*. Stations were located near the coast and within 1 km distance to each other (78.11°N 14.35°E, station 5; 78.10°N 14.38°E, station 6; 78.10°N 14.38°E, station 7; 78.10°N 14.39°E, station 23). Sampling dates were chosen according to light conditions: December 20th 2017 (24 h darkness), February 28th 2018 (7 h daylight), May 1st 2018 (24 h daylight), December 17th 2018 (24 h darkness), April 25th 2019 (24 h daylight), and September 13th 2019 (15 h daylight). Water depths varied between 3 and 9 m. (sample processing: Supplementary Information; all data: Supplementary Table S1).

### Sediment particle-size measurements

Particle-size measurements were performed in the Particle-Size Laboratory at MARUM, University of Bremen (Germany), with a Beckman Coulter Laser Diffraction Particle Size Analyzer LS 13320 (Beckman Coulter, Krefeld, Germany) following in principle the methodology of Boehnert et al. [33] (For details see Supplementary Information).

### Determination of carbon and nitrogen concentrations

Freeze-dried sediments were grounded to a powder in a Planetary Micro Mill (Pulverisette 7, Fritsch, Idar-Oberstein, Germany). To determine total carbon and nitrogen, ∼25–50 mg of sediment powder was packed in 5 × 9 mm tin capsules (HEKAtech, Wegberg Germany). To determine organic carbon, inorganic carbon was removed with 1 M HCl before analysis. Samples were analyzed in an Euro EA-CNS elemental analyzer (HEKAtech, column temperature 75 °C; carrier gas helium at 80 ml min^−1^, reactor temperature 1000 °C with oxygen flow 10 ml min^−1^, oxidation time 7.9 s) with thermal conductivity detection. Calibration was done with sulfanilamide (0.2–2 mg) standards. The quantification limit for carbon and nitrogen was 0.2 mg per gram sediment. In Helgoland samples, organic nitrogen concentrations were below quantification limit, hence values were not reported.

### Chlorophyll and phaeopigment measurements

Pigments were yielded by extraction from 1 ml sediment with 8 ml acetone and disruption in a cell mill. The supernatant was collected after centrifugation and the extraction of the sediment was repeated twice. Two milliliter aliquots of the pooled extracts were subjected to fluorescence measurements [34] using a Trilogy Laboratory Fluorometer (Turner Designs; excitation = 428 nm; emission = 671 nm) and measured before and after acidification with 100 µl 20% HCl. The concentration of chlorophyll *a* (Chl*a*), and phaeopigments (PhP) were determined according to

$$\begin{array}{*{20}{c}} {{\rm{Chla}}\left[ {{\mathrm{\mu }}{\rm{g}}} \right]} & = &\kern-1.0pc {({\rm{RFU}}_{\rm{b}} - {\rm{RFU}}_{\rm{a}}) \times CF \times AF \times f} \\ {{\rm{PhP}}\left[ {{\mathrm{\mu }}g} \right]} & = &\kern-0.5pc {({\rm{RFU}}_a \times {\rm{AR}} - {\rm{RFU}}_{\rm{b}}) \times CF \times AF} \end{array}$$

with the relative fluorescence units before acidification (RFU_b_) and after acidification (RFU_a_) and the calibration factor (CF) and the dilution factor (*f*) determined from chlorophyll standards as described by Lorenzen [34].

### DNA extraction, amplification of 16S rRNA genes, sequencing

DNA was extracted from sediment samples (0–2 cm depth) according to Zhou et al. [35], slightly modified by adding three initial freeze-thaw cycles. DNA from surface seawater was extracted using DNeasy Power Water Kit (QIAGEN, Hilden, Germany). Amplification of 16S rRNA gene fragments was done using primers S-D-Bact-0341-b-S-17 and S-D-Bact-0785-a-A-21 [36]. Amplicons were sequenced on an Illumina (San Diego, CA, USA) platform (HiSeq2500, 2 × 250 bases, paired-end) at the Max Planck-Genome Center in Cologne (Germany). Sequences were processed using BBTools version 37.62 [ref. 37], mothur v.1.38.1 [ref. 38] and classified using the SILVAngs pipeline and database SSU 138.1 Ref NR99 [ref. 39] (details: Supplementary information).

### Amplicon sequence variants (ASV) analysis

Demultiplexing and per sample extraction of forward and reverse fastq files was conducted using mothur v.1.39.5 ref. [38] and BBTools v. 37.90 ref. [37] ASVs were determined using dada2 v. 1.16.0 ref. [40] (standard pooled processing; SSU 138 Ref NR99). All ASV taxonomically classified as “Chloroplast”, “Archaea” or “Eukarya” as well as absolute singletons were removed from the dataset.

Alpha diversity was calculated using the subsamplingNGS.R function (https://github.com/chassenr/NGS/blob/master/Plotting/SubsampleNGS.R; 100 iterations) (For details see Supplementary information).

### Statistical analysis

Statistical tests were performed using the R package vegan [41–43] and customized R-scripts. ANOSIM analyses [44] were performed with 999 permutations at equal group size treatment.

### Separation of cells from sediment grains

Formaldehyde-fixed Svalbard sediment samples (for details see Supplementary Information) were sonicated on ice with a type MS2.5 probe (Sonoplus mini20; Bandelin, Berlin, Germany). Six sonication steps were done at a setting of 30 s, an amplitude of 86% and pulse of 0.2 s. Combined supernatants were filtered onto 0.2 µm pore size polycarbonate filters (GTTP, Millipore, Eschborn, Germany). More than 90% of all cells have been separated from the sand grains without obvious cell damage (Supplementary Fig. S1). Silty sediments of station 6 and 23 (September 2019) were sonicated only once, diluted and directly filtered.

### Catalyzed reporter deposition fluorescence in situ hybridization (CARD-FISH)

In situ hybridizations with horseradish peroxidase (HRP)-labeled probes followed by fluorescently-labeled-tyramide signal amplification were carried out as described previously [45], with few modifications. Inactivation of endogenous peroxidases was done by hydrogen peroxide (0.15% in methanol) for 30 min at room temperature. Hybridization was performed at 46 °C for 2–3 h in a chamber equilibrated with 2.25 M NaCl and identical formamide concentration as in the hybridization buffer. Amplification with Alexa 488-labeled tyramides was performed for 45 min at 46 °C. Filter sections were mounted with a mixture of CitiFluorAF1 (CitiFluor Ltd., London, United Kingdom] and Vectashield (Vector Laboratories, Burlingame, CA, USA) containing 1 μg ml^−1^ DAPI (4′,6-diamidino-2-phenylindole; Sigma-Aldrich, Steinheim, Germany). Probe sequences, permeabilization conditions and formamide concentrations are given in Supplementary Table S2.

### Automated imaging and counting

For cell counting of bacteria in Svalbard sediments, we improved an automated system that was established for bacterioplankton [46, 47]. Low signal to background ratios in sediments compared to seawater required the use of narrow band pass optical filter sets. Number of layers for the imaged z-stack was increased to 13 (distance 0.4 µm). Filter sections were imaged by microscopy using a motorized epifluorescence microscope (AxioImager.Z2m, Carl Zeiss, Jena, Germany) equipped with a 63×/1.4 Plan-Apochromat objective and controlled via the MPISYS software. Illumination was done using a Zeiss Colibri 7 LED source (385 nm for DAPI; 469 nm for Alexa488) using specific narrow band pass optical filter sets for DAPI (splitter 375 nm; emission filter 448/20 nm) and Alexa488 (splitter 495 nm; emission filter 520/15 nm). The images were analyzed using the Automated Cell Measuring and Enumeration tool 3.0 (ACMEtool) software (M. Zeder, www.technobiology.ch. [47]). (For technical details see Supplementary Information).

### Probe design

Probe design for Actinobacteriota based on SILVA database SSU 132 Ref NR99 and was performed with the probe design tool implemented in ARB [48] (Supplementary Fig. S2). CARD-FISH with a mix of probes ACM1218 (AGCATGCGTGCAGCCCTG; Actinomarinales) and MIT1218 (AGCATGTTTGCAGCCCTG; Microtrichales) resulted in counts that were equal to the sum of individual counts (92 ± 9%) confirming high probe specificity. An increase of probe concentration to 280 fmol µl^−1^ (10-fold above standard conditions) resulted in brighter signals and higher cell numbers (1–3-fold increase for ACM1218; 2–6-fold increase for MIT1218).

### Actinobacteriota imaging, cell size, and cell division

Actinobacteriota were imaged by a laser scanning microscope (LSM780, Zeiss, Jena, Germany) equipped with an Airyscan detector. For cell size measurements, FISH [49] with Alexa594-tetralabelled probes and CARD-FISH hybridization buffer was used to avoid an overestimation of cell sizes by too bright CARD-FISH signals.

The fraction of dividing cells was determined based on line profiles of DAPI and FISH signal fluorescence in formaldehyde-fixed Actinomarinales cells (Supplementary Fig. S3). Dividing cells were defined by two criteria: i) two DAPI maxima are visible in a single cell and ii) one or no FISH signal maximum is present in the cell center between the two DAPI maxima. If two FISH signal maxima were detected in the cell center, cell division was categorized as completed.

## Results

### Grain size

**Fig. 2:**
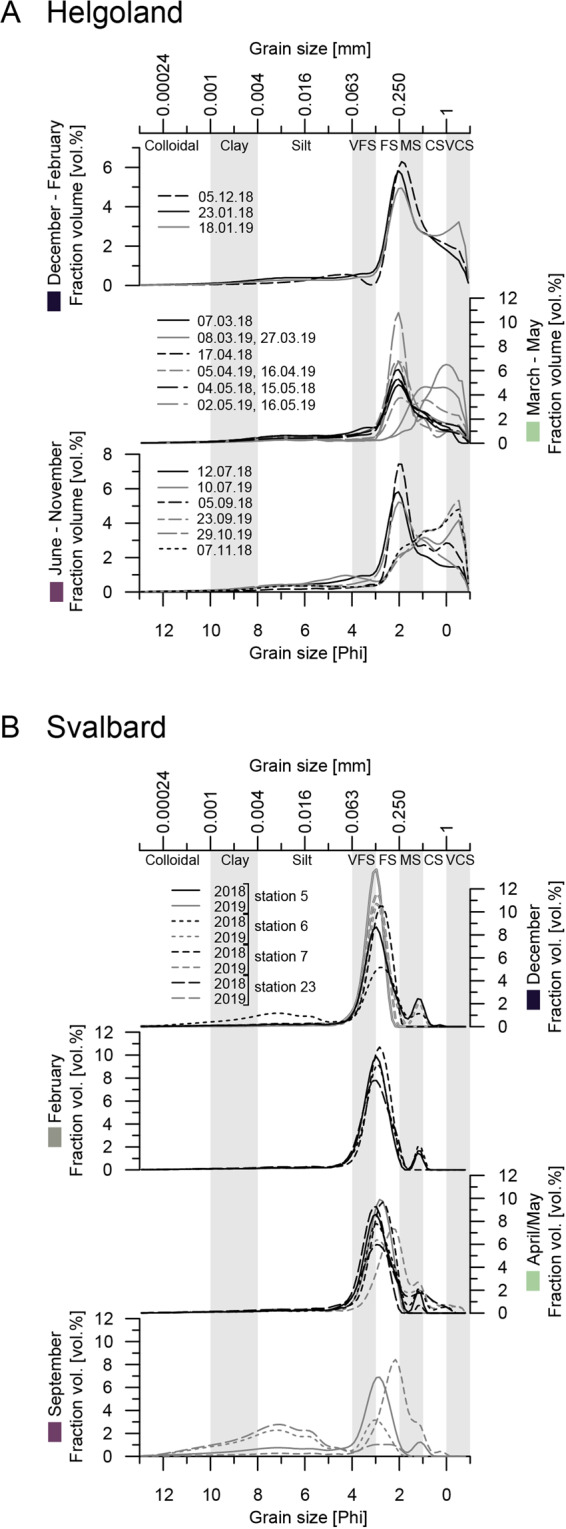
Grain size distribution (vol%) of A Helgoland and B Svalbard surface sediments (0–2 cm depth) derived from laser diffraction particle size analysis. Please note the different y-scales for Helgoland samples.

Helgoland surface sediments were, in general, composed of fine and medium sand with variable portions of coarse to very coarse sand (Fig. 2A). Winter and early spring sediments contained a large portion of coarse to very coarse sand. Svalbard sediments of Isfjorden were mainly composed of very fine and fine sand with a small fraction of medium sand. Grain size distribution did not differ markedly across stations and seasons (Fig. 2B). Except for station 7, grain size distribution changed in September 2019. Sediments from station 5 contained a minor portion of silt in addition to fine sand and medium sand. Composition of stations 6 and 23 sediments was shifted towards silty sand (station 6) and sandy silt (station 23).

### Organic carbon and nitrogen contents

**Fig. 3:**
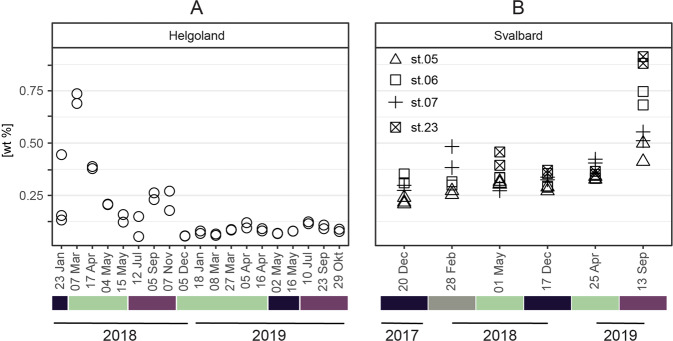
Total organic carbon content in **A** Helgoland and **B** Svalbard sediments. Seasons are color-coded (winter: black, twilight: gray; spring: green; summer/autumn: aubergine-colored).

In Helgoland sediments, total organic carbon (TOC) content varied between 0.1% and 0.7% of sediment dry weight in 2018 (Fig. 3A). In 2019, TOC was constantly low (<0.13%). These TOC contents were typical for surface sediments of the Southern North Sea (<0.1 mg to 10 mg g^−1^, see ref. [50]) In Svalbard sediments, TOC content was between 0.2% and 0.5% in the winter, twilight, and spring samples (mean 0.3 ± 0.1%; Fig. 3B). In September 2019, TOC in silty sediments from station 6 and 23 was highest with 0.7 and 0.9%.

Organic nitrogen content in Svalbard sandy sediments was relatively stable (Supplementary Fig. S4), with quantities between 0.03 to 0.08% of sediment dry weight for all seasons. Total organic carbon/organic nitrogen (TOC/*N*_org_) ratios were 9.8 ± 0.9. Organic nitrogen contents of 0.08 ± 0.01% were detected in silty sediments from stations 6 and 23 in September 2019. TOC/*N*_org_ ratio was 11.2 ± 0.4. TOC, *N*_org_, and TOC/*N*_org_ ratios changed with grain size. In silty sands TOC was 0.8 ± 0.1%, *N*_org_ 0.1 ± 0.01% and a TOC/*N*_org_ ratio of 11.1 ± 0.4, while sandy sediments had a significantly lower TOC of 0.3 ± 0.08%, lower *N*_org_ of 0.04 ± 0.01% and a slightly lower TOC/*N*_org_ ratio of 9.8 ± 0.9.

### Chlorophyll *a* concentrations

In Helgoland sediments, concentrations of chlorophyll *a* ranged between 0.7 and 5.3 µg ml^−1^ (average 1.9 ± 1 µg ml^−1^) and concentrations of phaeopigments between 0.1 to 1.3 µg ml^−1^ (average 0.4 ± 0.3 µg ml^−1^; Supplementary Table S1). On 4th of May 2018, we detected the strongest chlorophyll *a* peak (5.2 µg ml^−1^) while concentration at all other time points were 2.6 µg ml^−1^ and lower. Nevertheless, no clear increase of chlorophyll concentrations in sediment samples from spring was observed.

In Svalbard sediments, chlorophyll *a* concentrations were very low in winter and twilight samples (0.3 ± 0.1 µg ml^−1^ and 0.2 ± 0.1 µg ml^−1^) and clearly higher in spring 2018 and summer/fall (1.2 ± 0.5 µg ml^−1^ and 1.1 ± 0.6 µg ml^−1^). In spring 2019, we measured only 0.3 ± 0.1 µg chlorophyll *a* per ml of sediment. At the same time, however, chlorophyll *a* concentrations in surface seawater were high by now (7 ± 1 µg l^−1^) compared to winter 2018 (0.4 ± 0.02 µg l^−1^, Supplementary Table S1).

### Bacterial community composition

The composition of bacterial communities of Helgoland and Svalbard surface sediments (0–2 cm depth) and Svalbard surface seawater was investigated by 16S rRNA gene sequencing. For simplicity, Helgoland sampling dates were assigned to meteorological seasons. Svalbard sampling dates were assigned to seasons based on light availability: winter (December 2017, 2018), twilight (February 2018), spring (April 2019, May 2018), and summer/fall (September 2019). Numerous 16S rRNA gene sequences from chloroplasts were detected in the dataset from both, Helgoland and Svalbard. In Svalbard sediments, they constituted 4 ± 2, 11 ± 7, 36 ± 26, and 31 ± 11% of total reads and in surface seawater 1 ± 0.2, 3 ± 0.3, 80 ± 10, and 26 ± 3% of total reads in winter, twilight, spring, and summer/fall, respectively. The strongly increased read frequency of chloroplast sequences in datasets of spring samples indicated the presence of a current or recent phytoplankton bloom. In Helgoland sediments portions of 16S rRNA chloroplast sequences were low for all seasons. They constituted only 4 ± 2, 3 ± 2, and 4 ± 2% of total reads during winter, spring, and summer/fall samples.

#### Helgoland sediments

**Fig. 4:**
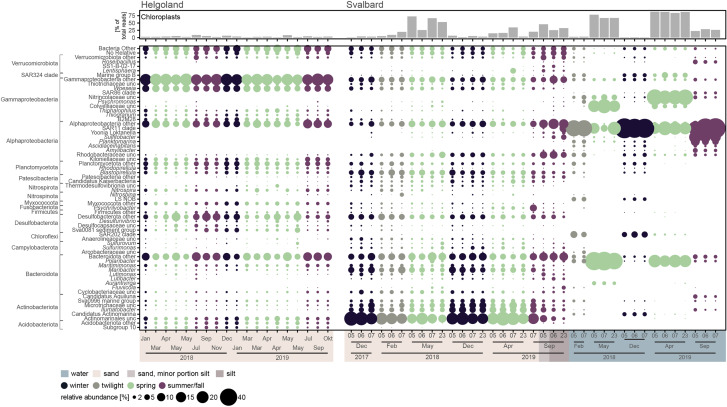
Relative abundance of bacterial families and genera in Helgoland surface sediments and Svalbard sediments and seawater based on sequencing of 16S rRNA genes (V3-V4 region). Taxonomy based on SILVA SSU138.1 Ref NR99 database. Only taxa that accounted for >2% of total sequences in at least one of the samples are shown. Minor abundant taxa were clustered on higher taxonomic levels and displayed as “other”. The bubbles’ diameters give read frequencies, bubbles’ colors indicate the season of sampling. For Svalbard samples, station numbers are indicated on the *x*-axis. As proxy for current or recent photosynthetic activity and primary production, read frequencies of sequences classified as chloroplasts are shown in the top bar charts.

The bacterial composition was stable for all 19 sampling dates (Fig. 4). The community was dominated by Gammaproteobacteria (winter: 35 ± 2% of reads, spring: 34 ± 4%, summer/fall: 32 ± 3%). Within this class, *Woeseia* spp. dominated and did not change abundance (winter, spring, and summer/fall: 7 ± 1%). Further taxa with high read frequencies were Planctomycetota (winter: 8 ± 0.4%, spring: 7 ± 1%, summer/fall: 7 ± 1%), Alphaproteobacteria (winter: 11 ± 2%, spring: 10 ± 3%, summer/fall: 12 ± 3%), Desulfobacterota (winter: 7 ± 3%, spring: 10 ± 4%, summer/fall: 9 ± 4%), and Bacteroidota (winter: 11 ± 3%, spring: 11 ± 3%, summer/fall: 14 ± 2%).

#### Svalbard sediments

The bacterial community composition showed no clear changes between seasons (Fig. 4). Note that for calculation of mean read frequencies, data from silty sediments (station 6 and 23, September 2019) were excluded to avoid influence of reduced permeability and oxygen availability due to smaller grain size. Across all seasons, Actinobacteriota dominated the benthic community with 29 ± 9% of reads (winter: 34 ± 1%, twilight: 26 ± 1%, spring: 31 ± 8%, summer/fall: 25 ± 11%). These Actinobacteriota were mainly affiliated with uncultured Actinomarinales (17 ± 7% of reads; winter: 20 ± 4%, twilight: 17 ± 2%, spring: 18 ± 6%, summer/fall: 12 ± 6%) and Microtrichales (12 ± 4% of reads; winter: 14 ± 4%, twilight: 8 ± 2%, spring: 13 ± 3%, summer/fall: 12 ± 5%). Other abundant taxa were Bacteroidota (winter: 19 ± 1% of reads, twilight: 18 ± 2%, spring: 18 ± 3%, summer/fall: 20 ± 4%) with *Maribacter* being most dominant (4 ± 2%) and Planctomycetota (winter: 7 ± 2%, twilight: 9 ± 2%, spring: 6 ± 2%, summer/fall: 5 ± 0.2%). Stable read frequencies were also detected for diverse Proteobacteria: Gammaproteobacteria (winter: 11 ± 1%, twilight: 13 ± 2%, spring: 11 ± 2%, summer/fall: 8 ± 1%), Alphaproteobacteria (winter: 9 ± 3%, twilight: 8 ± 1%, spring: 10 ± 3%, summer/fall: 16 ± 8%), and Desulfobacterota (winter: 5 ± 1%, twilight: 7 ± 1%, spring: 4 ± 1%, summer/fall: 4 ± 0.3%). ANOSIM statistics indicated that observed differences in read frequencies between seasons were not significant (*R* = 0.15, *p* = 0.09).

In the two silty sediments (station 6 and 23, September 2019), communities were characterized by lower read frequencies of Actinobacteriota (9 ± 2% vs. 29 ± 9% in sandy sediments) and *Maribacter* (0.6 ± 0.1% vs. 4 ± 2%) and by higher frequencies of alphaproteobacterial Rhodobacteraceae (20 ± 1% vs. 8 ± 5%). (Fig. 4). These changes were significant (Supplementary Fig. S5). Further significant changes were detected for less abundant genera of Bacteroidota, i.e., *Maritimonas*, *Maribacter*, *Lutibacter*, and *Fluviicola*.

#### Svalbard seawater

Bacterial communities in Svalbard surface seawater showed distinct seasonal patterns (Fig. 4). In winter, twilight and summer/fall, SAR11 dominated (53 ± 13% of reads). In spring, SAR11 decreased to 23 ± 8% while genus *Polaribacter* increased from 2 ± 1 to 31 ± 12%. Further taxa that strongly increased in spring 2019 were Nitrincolaceae (30 ± 4% vs. 1 ± 1% across other seasons) and Colwelliaceae in spring 2018 (23 ± 5% vs. 0.1 ± 0.2%).

#### Comparison of Svalbard and Helgoland sediment communities

**Fig. 5:**
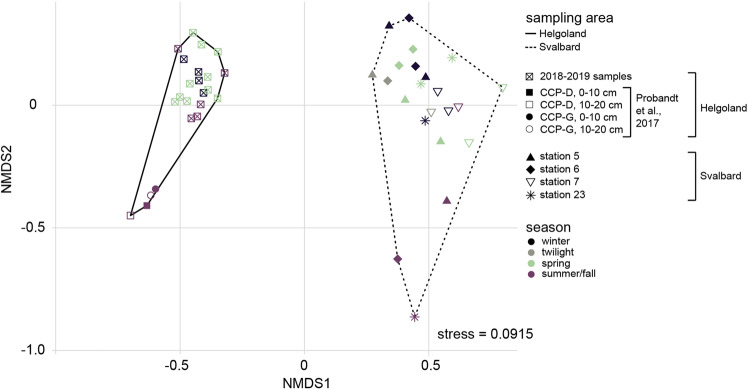
NMDS ordination plot of sampling areas at Helgoland and Svalbard, generated from 16S rRNA gene datasets. Data from sites CCP-D and CCP-G, 6.5 km apart from Helgoland (bioproject number PRJEB18774, ref. [28]) were included in the analysis. The sampling areas are depicted as polygons. Helgoland and Svalbard bacterial community structures were significantly different supported by an ANOSIM *R* value of 0.96 (*p* < 0.0001). Dissimilarity based on season was not supported by ANOSIM (*R* = 0.06, *p* = 0.09).

Even though Helgoland and Svalbard benthic bacterial communities were seasonally stable, they harbored significantly different communities (ANOSIM; *p*<0.0001, *r* = 0.963; Fig. 5). Read frequencies of Actinobacteriota, *Maribacter*, and Patescibacteria were much lower at Helgoland than at Svalbard (Actinobacteriota, 8 ± 1% vs. 31 ± 6% of reads; *Maribacter*, 0.7 ± 0.2% vs. 5 ± 2%; Patescibacteria, 0.4 ± 0.1% vs. 3 ± 1%). In contrast, Acidobacteriota, were more abundant in Helgoland sediments (3.5 ± 0.4% vs. 0.6 ± 0.1%). Different quantities and compositions of organic matter depending on the characteristics of the bloom as well as different grain size distributions might have caused the differences.

### In situ quantification of major taxa in Svalbard sediments

In situ quantification of major taxa focused on the region where we expected the most prominent seasonality, Svalbard. As a prerequisite, we modified the automated system developed for cell enumeration of bacterioplankton [46, 47]. This system worked for both types of Svalbard sediments, sand and silt. Manual counts constituted 89 ± 10% of automated counts (DAPI-stained cells), 112 ± 26% (Desulobacterota/Myxococcota, probe DELTA495a-c) and 98 ± 21% (Actinomarinales, probe ACM1218) (Supplementary Fig. S6). Although this system did not greatly reduce the time for sample processing compared with manual cell counting, it allows more standardized, thus more reliable, cell counting, and exclusion of human bias.

Total cell numbers varied between 1.2 × 10^8^ and 1.3 × 10^9^ cell ml^−1^ sediment (Supplementary Fig. S7). To normalize the differences in total cell counts, we focused on relative cell numbers to identify changes between seasons.

**Fig. 6:**
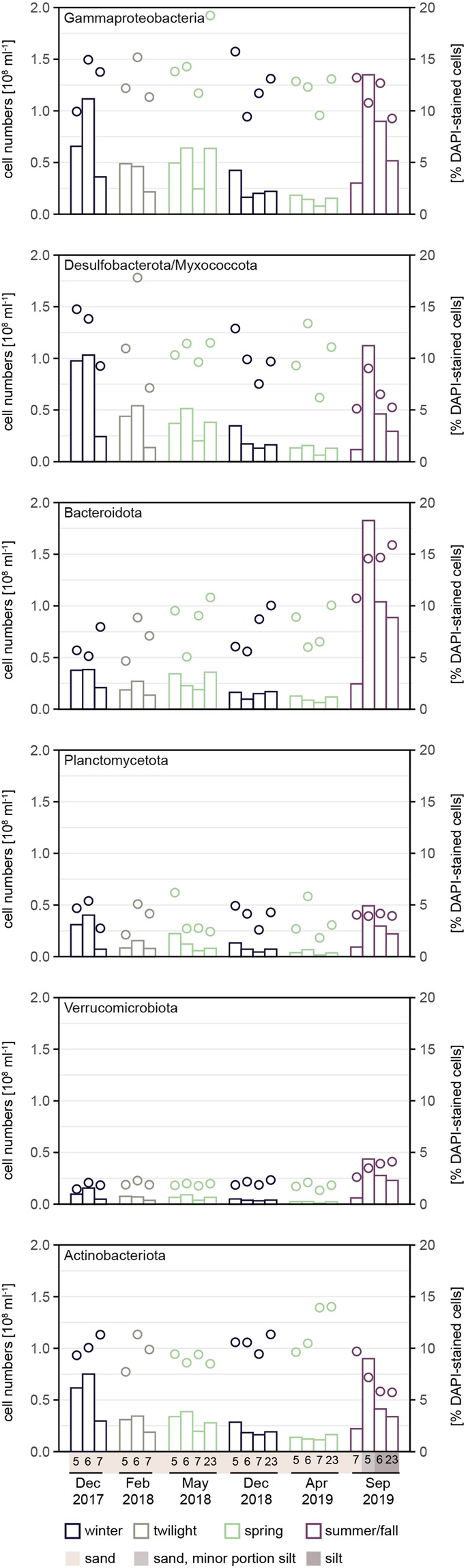
In situ abundance of bacterial taxa in Svalbard sediments as determined by CARD FISH. Bars give absolute cells numbers, circles give relative abundances. Seasons are color-coded (winter: black, twilight: gray; spring: green; summer/fall: aubergine-colored).

Overall, relative cell numbers of major taxa did not change significantly between winter and spring (*t*-test: *p*-values between 0.260 and 0.842) (Fig. 6): Gammaproteobacteria (winter: 13 ± 2%, twilight: 13 ± 2%, spring: 13 ± 3%, summer/fall 12 ± 2%), Desulfobacterota/Myxococcota (winter: 11 ± 3%, twilight: 12 ± 5%, spring: 10 ± 2%, summer/fall 7 ± 3%), Planctomycetota (winter: 4 ± 1%, twilight: 4 ± 2%, spring: 3 ± 2%, summer/fall 4 ± 0.1%) and Verrucomicrobiota (winter: 2 ± 0.3%, twilight: 2 ± 0.2%, spring: 2 ± 0.2%, summer/fall 3 ± 0.6%). Actinobacteriota of Actinomarinales and Microtrichales were found in high numbers, up to 10 ± 2% of total cells across all seasons (Supplementary Fig. S7). Actinomarinales made up 5 ± 1% of total cells (winter: 6 ± 1%, twilight: 5 ± 1%, spring: 6 ± 1%, summer/fall 5 ± 2%) and Microtrichales 4 ± 1% (winter: 5 ± 1%, twilight: 5 ± 1%, spring: 5 ± 1%, summer/fall 4 ± 0.3%).

Across seasons, the only striking change in relative cell numbers was detected for Bacteroidota, which increased by a factor of ∼2 in summer/fall (winter: 7 ± 2%, twilight: 7 ± 2%, spring: 8 ± 2%, summer/fall 13 ± 3%). The change between winter and summer/fall was significant (*t*-test, *p* = 0.010), however, we refrain from linking it to season because station 5 contained a minor part of silt besides fine sand, resulting in reduced permeability and oxygen availability (oxygen saturation in sediments from station 5 was lower than at station 7, data not shown). The influence of sediment-type on the bacterial community is even more pronounced in silty sediments of summer/fall where Bacteroidota increased to 15 ± 1% of total cells. Our data support previous findings that permeability shapes the bacterial community [51, 52].

### Amplicon sequence variants and diversity parameters

On the taxonomic level of genus and higher, Svalbard benthic bacterial communities did not show significant seasonal changes based on CARD-FISH cell numbers and read frequencies (Figs. 4 and 6). To address possible changes below genus level, we investigated seasonal changes of amplicon sequence variant (ASV) frequencies (Supplementary Table S3). In sediments, variations in frequencies of overall most abundant ASV between spring and winter were all within standard deviation (of the eight samples per category) indicating no significant change (Supplementary Fig. S8). Variations in frequencies of ASV from Gammaproteobacteria, Desulfobacterota, Bacteroidota, Verrucomicrobiota, and Actinobacteriota were also within standard deviation (Supplementary Fig. S9 and Supplementary Table S3). In contrast, frequencies of seawater ASV changed significantly and showed variations by factors of 6 and 1928 between spring and winter for the ten most abundant ASV.

Alpha diversity analysis as given by Inverse Simpson showed a lower diversity in Svalbard sediments from winter (131 ± 36) compared to spring (188 ± 62), however, the differences were still within standard deviation (Supplementary Table S4). In seawater, Inverse Simpson were higher in winter (16 ± 2) than in spring (12 ± 5), mirroring the pelagic bacterial community dynamics detected by ASV frequencies.

### Actinobacteriota

Actinobacteriota were the major phylum in Svalbard sediments based on cell numbers and read frequencies. The major part of Actinomarinales sequences was affiliated with clades Uncultured 1 and Uncultured 6 (Supplementary Fig. S2) that comprise sequences from diverse marine habitats (for example, Mediterranean beaches [53], Arctic sediments [54], polar waters [55], seafloor lavas [56]). Most abundant ASV#473 (2.6% of total reads) and ASV#839 (1.8%; Supplementary Table S3) were both affiliated with clade Uncultured 6. Microtrichales sequences were mainly affiliated with *Ilumatobacter*, clade Sva0996, and clade Uncultured 10. Sva0996 was first found in Svalbard silty sediments from Hornsund [57]. Nowadays, Sva0996 comprises sequences from numerous marine habitats including beaches [53], permeable shelf sediments [58], tidal subsurface sediments [59], or seawater [60].

**Fig. 7:**
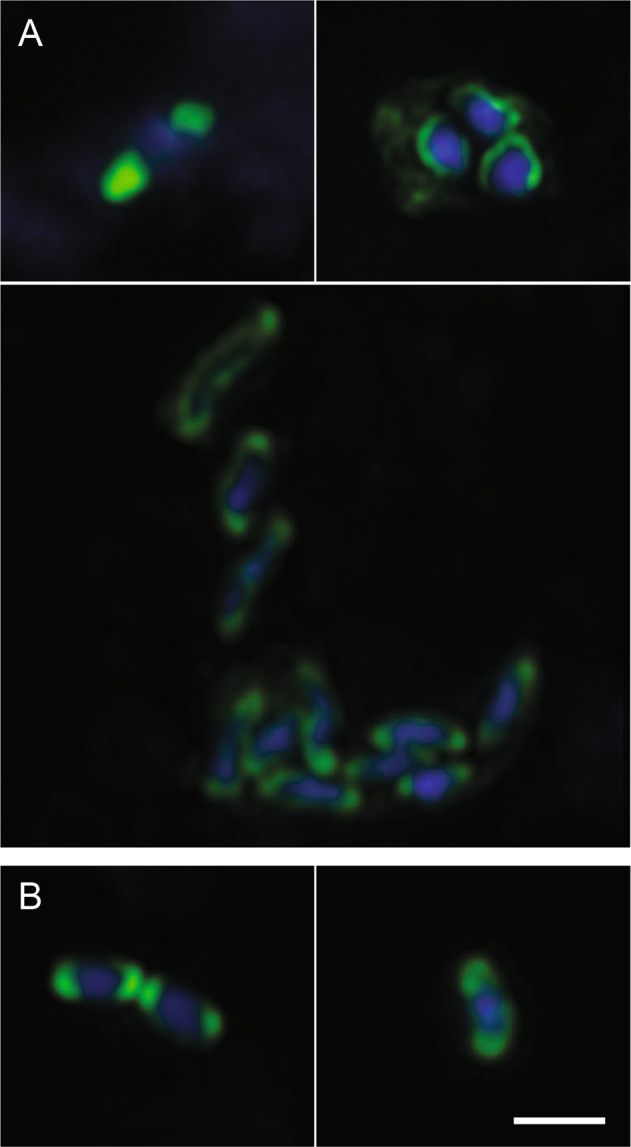
Laser scanning micrographs of Actinobacteriota in Svalbard sediments as detected by CARD-FISH. **A** Actinomarinales visualized by probe ACM1218; **B** Microtrichales visualized by probe MIT1218. Green, CARD-FISH signals; blue, DAPI signals. Scale bar, 1 µm.

In situ detection revealed ∼95% of Actinomarinales were rods while ∼5% were cocci. Most cells were free-living, few cells occurred in loose biofilms (Fig. 7A). Microtrichales were also rod-shaped (Fig. 7B), few cells were coccoid (∼10%). Based on high-resolution images, Actinomarinales were highly active as indicated by a frequency of dividing cells of 28% (station 6, December 2017; 78 cells analyzed; Supplementary Fig. S3). After division, Microtrichales and Actinomarinales cells had a size of approximately 0.25 × 0.5 µm and a volume of 0.024 µm^3^.

## Discussion

### Benthic bacterial communities are stable across seasons

A major part of the annual primary production in temperate and high latitude environments occurs during spring [30, 61–63]. Especially in the Arctic, the amounts and relative contributions of the different organic matter sources to fjords change seasonally and spatially [63–65]. Light is the limiting factor for phytoplankton growth during winter. In addition to light availability, runoff of suspended sediment from glaciers in summer and fall drives seasonality of organic matter input [66]. In Isfjorden, marine organic matter dominates in May following the spring phytoplankton bloom while in June and August, permafrost and glacial-fed meltwater is present and a source of terrestrial organic matter [66]. Surface sediments at our sampling sites in Isfjorden are located in a wave-dominated embayment and are thus little affected by meltwater and river runoff (Rubensdotter and Jensen, 2020, map of Hollendarbukta stored at repository: https://svalcoast.com/). This is also indicated by only a minor shift in TOC/*N*_org_ between winter (10.0 ± 0.5) and spring (9.5 ± 1.0) and summer/fall (10.8 ± 0.5), although glacial meltwater runoff is probably the reason for more silty samples in September. In the bacterioplankton community, the seasonality in primary production at Svalbard was clearly causing compositional changes (Fig. 4 and Supplementary Fig. S8) such as a strong increase of *Polaribacter* and *Aurantivirga*, as previously seen also for Helgoland seawater [18, 19, 21, 67]. However, the benthic bacterial communities at Svalbard and Helgoland, remained stable over two annual cycles. Cell numbers of major taxa and read frequencies of 16S rRNA genes on genus-level and below did not change significantly between winter and spring. Increased read frequencies of chloroplasts and chlorophyll concentrations in spring 2018 samples and summer/fall 2019 samples (Fig. 4 and Supplementary Table S1) indicated, that we did not miss the phytoplankton bloom or fresh organic matter from benthic primary production. Thus, the bacterial community might have responded to phytodetritus input by an increased activity rather than by changes in community structure. A response dominated by increased activity is supported by results of recent in situ experiments at Fram Strait, part of the Arctic deep-sea floor, where phytodetritus of either the diatom *Thalassiosira* sp. or the coccolithophore *Emiliania huxleyi* was provided to the benthic community. The community did not respond to the algal input by an increase of total bacterial cell numbers, but by an increase of exoenzymatic activities [68]. The high diversity of Svalbard benthic bacterial communities (observed number of ASV between 2526 and 6691) is coincident with a diverse spectrum of enzymes capable of degrading different substrates as previously suggested by Teske and colleagues [15]. Thus, responses to substrate input might preferentially be on the gene expression level rather than in community structure.

Although major taxa such as Actinomarinales were actively growing in Svalbard sediments, as shown by a high frequency of dividing cells (28%), we did not detect an increase in absolute cell numbers in any season. Bacterial biomass turnover time is short, typically within 2–18 days in organic-poor sands (average 5–6 days [11]). Grazing, however, is not considered the major process controlling the fate of bacterial biomass [69, 70]. By an in situ pulse-chase experiment with ^13^C-tracers, van Oevelen and colleagues [70] quantified the fate of microphytobenthos in an estuarine tidal flat. They observed that grazing on bacteria by higher trophic levels is limited to 9% of total bacterial production. Virus-induced prokaryotic mortality account for 16 ± 3% in coastal sediments (*n* = 11) [71].

In addition to loss via grazing and viruses, the seasonal stability of major taxa may well be linked to their physical growth conditions on sand grains. Life in cracks and depressions of the sand grain may protect microbes from grazing and abrasion when sand moves [72]. Microbes are neither evenly distributed nor do they form thick biofilms on sand grains: they colonize only about 4% of the grains’ surface area [52, 73]. Non-populated, convex areas on the sand grains’ surface could be too exposed to grazers and shearing forces during sediment reworking to maintain microbial populations. Large depressions might be diffusion-limited, reducing oxygen and nutrients supply to microbes [73]. Thus, our observations of i) active growth and ii) lack of seasonality of bacterial communities even after phytodetritus-input together with generally low population density on sand grains suggests a maximum of habitable surface area on a sand grain that keeps the bacterial community structure in a steady-state.

### Actinobacteriota are key heterotrophs in Svalbard sandy surface sediments

Actinobacteriota of uncultured Actinomarinales and Microtrichales clades constituted the major taxon in Svalbard and Helgoland sandy surface sediments (Figs. 4 and 6). Taking into account the high read frequency of *Ilumatobacter* (5%) and the missing coverage of this genus by the probes, the detected actinobacterial in situ abundance of 10% of total cells is likely still underestimated. Actinobacteriota constitute one of the most diverse bacterial phyla [74]. For decades, they were mostly viewed as soil bacteria, however, nowadays they are considered to be cosmopolitan in terrestrial and marine ecosystems [75]. Among marine bacterioplankton communities, Actinobacteriota are ubiquitous [76] and abundant (16S rRNA read frequencies ~10%) [77]. Pelagic and deep-sea benthic Actinobacteriota (16S rRNA read frequencies 4–31%) [78] mainly belong to “*Candidatus* Actinomarina”, also known as OM1 [79], which was only rarely detected (<1%) in our datasets. In other Arctic surface sediments, actinobacterial sequences were either absent [15] or found in moderate (5% refs. [57, 80]) and high abundances (4–20% of total reads [81]).

Studies on marine Actinobacteriota focused mainly on secondary metabolite production [82–84], thus their metabolism is largely unknown. In general, most Actinobacteriota are aerobic chemoheterotrophs using a wide variety of complex organic matter, including cell wall polysaccharides of plants such as cellulose, xylan, mannan, and other hemicelluloses, chitin or humic substances (for a review see ref. [85]). Actinobacteriota encode a huge diversity of carbohydrate-active enzymes. For example, fourteen glycoside hydrolases including GH3, GH18, GH36, GH65, and GH92 were found in the *Ilumatobacter coccineum* genome [86]. Based on the expression of a new kind of rhodopsin, a photoheterotrophic lifestyle was suggested for *Candidatus* Actinomarina [87]. Light, however, is not a key factor determining in situ abundance and activity of uncultured Actinomarinales in Svalbard sediments, as 28% were dividing in winter samples and cell numbers did not differ between seasons. Considering the high frequency of dividing actinobacterial cells and their high in situ cell numbers of at least 10% of total microbial community, uncultured Actinomarinales and Microtrichales likely play an important role in carbon mineralization in Svalbard sediments. Based on the carbohydrate-active enzymes typical of Actinobacteriota [85, 88, 89], we suggest that Actinomarinales and Microtrichales may be key heterotrophs hydrolyzing complex substrates.

## Conclusions and outlook

Pelagic and benthic microbial communities respond to phytoplankton productivity very differently. While the bacterial community of the water column dynamically responds to seasonal substrate input in cell numbers and composition, we observe stable benthic communities throughout two years of sampling. Thus, our hypothesis that substrate-based seasonality extends to benthic bacterial communities on the level of cell counts and 16S rRNA-based taxonomy has been falsified for polar sediments from Svalbard as well as for temperate sediments from Helgoland.

Further studies must now target metagenomics and metatranscriptomics, metabolic activity, and gene regulation. For example, expression of glycoside hydrolases of GH16 (laminarinase) is expected to vary strongly between seasons, as the sugar polymer laminarin is a major storage compound in marine microalgae [90]. In contrast, animal-derived glycoproteins such as mucin are expected to be equally abundant throughout the year [91], and the respective degradative enzymes should be available during all seasons. The seasonal utilization of different substrates could be tested by applying fluorescently-labeled substrates [92], and this could be combined with identification of specific polysaccharide utilization loci and their expression [93].

Actinomarinales and Microtrichales should be target taxa in future studies. The likely absence of an outer membrane in these actinobacterial cells would not allow for polysaccharide degradation by selfish uptake, a major mode in the water column [94]. Previous studies showed relatively high extracellular polysaccharide hydrolysis rates of ∼0.5 to 6 nmol monomer cm^−3^ h^−1^ in Svalbard sediments [95, 96]. Thus, we propose selfish uptake being of minor importance in sediments while degradation with exoenzymes dominates, releasing small substrates utilized by the plethora of microbial taxa living on sand grains.

## Supplementary Information

Supplemental Material

Supplementary Table S3

## Data Availability

Sequence data were stored in the European Nucleotide Archive (ENA) under study accession numbers PRJEB42060 and PRJEB42159. Other data from this study are available from the data repository PANGAEA (https://doi.pangaea.de/10.1594/PANGAEA.930322, https://doi.pangaea.de/10.1594/PANGAEA.930483, https://doi.pangaea.de/10.1594/PANGAEA.930442).
