# Supplementary material for: Bacterial communities in temperate and polar coastal sands are seasonally stable
Source: ISME Commun. 2021 Jun 28;1:29. doi: 10.1038/s43705-021-00028-w (PMC9723697; doi:10.1038/s43705-021-00028-w)
Supplement: Supplementary file 1 — Supplemental Material [file 43705_2021_28_MOESM1_ESM.pdf]

Supplementary Information

Bacterial communities in temperate and polar coastal sands are seasonally stable

Sebastian Miksch, Mirja Meiners, Anke Meyerdierks, David Probandt, Gunter Wegener, Jürgen Titschack, Maria A. Jensen, Andreas Ellrott, Rudolf Amann, Katrin Knittel

Supplementary materials and methods

Sample processing

Helgoland. The cores were kept at in situ temperature for about 30 minutes until processing. The sediment surface layer of 0-2 cm depth was homogenized and frozen at -20°C for later DNA extraction.

Svalbard. Subsampling and processing of sediment was done on board directly after retrieval. The surface layer of 0-2 cm depth was homogenized and frozen on dry ice for subsequent DNA extraction. For FISH and cell counting, 0.5 g of sediments was fixed with 1.3% formaldehyde (final concentration) for up to 6 h at room temperature, washed twice with 1x phosphate-buffered saline (PBS; 10 mM sodium phosphate, pH 7.2, 130 mM NaCl), and stored in 1xPBS–ethanol (1: 1) at -20°C. A volume of 300 ml of surface seawater was filtered through a 0.22 µm pore size polyethersulfone membrane filter (Millipore Sterivex-GP filter unit, Merck, Sigma-Aldrich, Munich, Germany) that was frozen on dry ice.

Sediment particle-size measurements.

Particle-size measurements were performed in the Particle-Size Laboratory at MARUM, University of Bremen (Germany), with a Beckman Coulter Laser Diffraction Particle Size Analyzer LS 13320 (Beckman Coulter, Krefeld, Germany). Prior to the measurements, the samples were boiled with ~0.3 g tetra-sodium diphosphate decahydrate ($Na_4P_2O_7 \times 10H_2O$, 3 min) to destroy aggregates (see also [1]). Sample preparation and measurements were carried out with deionized, degassed and filtered water (filter mesh size: 0.2 µm) to reduce the potential influence of gas bubbles or particles within the water. The reproducibility was checked regularly by replicate analyses of three internal glass-bead standards and was found to be better than ±0.7 µm for the mean and ±0.6 µm for the median particle size (1σ). The average standard

deviation integrated overall size classes is better than ±4 vol% (note that the standard deviation of the individual size classes is not distributed uniformly).

PCR amplification

For amplification of 16S rRNA gene fragments, two (Helgoland) and four replicate PCR reactions (Svalbard) were performed for each sample. The reactions contained 1x TaKaRa buffer, 0.2 mM dNTPs, 0.5 µM of each primer S-DBact-0341-b-S-17 and S-D-Bact-0785-a-A-21,[Ref.2] 1.875 U μl^{-1} Ex Taq DNA Polymerase (Takara Bio, Saint Germain-en-Laye, France) and ~10 ng DNA. The PCR program started with an initial denaturation step for 2 min at 95 °C, followed by 36 cycles of 94 °C for 1 min, 55 °C for 1 min, 72 °C for 2 min and a final extension step for 10 min at 72 °C. For amplification of gene fragments from seawater DNA, TaKaRa Taq DNA Polymerase was used. Amplicons were purified using Agencourt AMPure XP beads at a beads:DNA ratio (v/v) of 0.8 (Beckman Coulter, Krefeld, Germany). Equimolar amounts of the amplicons were pooled and size-selected (470 ± 50 bases) using a BluePippin (Sage Science**,** Beverly, MA, USA).

Quality trimming and sequence processing.

Reads were checked for adapter content and sequence quality using FastQC v0.11.4.[Ref3] Paired reads were merged using BBMerge of BBTools version 37.62[Ref.4] using standard parameters, with the exception useoverlap=t, merge=t and mismatches=0. Using mothur v.1.38.1,[Ref.5] trim.seqs was applied to remove barcodes and forward primer and to demultiplex the data. Afterwards the remaining sequences were reverse complemented using reverse.seqs and checked again using trim.seqs. The two output files were combined. Standard conditions were chosen for both commands, except trim.seq command. Qthreshold=15, allfiles=T and checkorient=f.

Sequences were randomly subsampled to 20,000 per sample for Helgoland and Svalbard datasets and were classified using the SILVAngs pipeline and database SSU138.1 Ref NR99.[Ref.6] The pipeline was run under default settings, resulting in a global operational taxonomic unit (OTU) clustering at 98% sequence similarity. More than 98% of submitted sequences could be classified by the SILVAngs pipeline. Absolute singletons as well as sequences classified as Archaea, Eukarya, and chloroplasts were removed prior to community composition analysis. Abundance tables were imported into R version 3.6.0.[Ref.7] Data processing and visualization was done using the package tidyverse.[8]

To display sample ordination based on the taxonomic fingerprint of the community, relative abundances of all classified taxa was used to calculate an ordination based on Bray Curtis dissimilarity using the metaMDS() function from the vegan R-Package and displayed using ggplot2. Besides all sediment samples from this study sequence information from a related study by Probandt *et al.* 2017 was included. Hierarchical clustering of samples was achieved using Bray Curtis dissimilarity calculated by the vegdist() function (vegan package) and clustered by using the hclust() function. Further information of several transformation steps can be found in the script.

Script is deposited at:

https://github.com/smiksch/16_S_rRNA_processing_looking_at_seasonality_in_sediments

Amplicon sequence variant (ASV) analysis.

Raw read fastq-files were split into fasta and qual files using reformat (BBTools v 37.90), demultiplexed using the trim.seqs function of mothur v. 1.39.5[Ref.5] (bdiffs=0, pdiffs=0, allfiles=T, flip=F, maxambig=0, maxhomop=10, checkorient=F), headers were extracted from demultiplexed fasta-files using the list.seqs function of mothur, and for each sample forward and reverse fastq files with reads in matching order were extracted using filterbyname

(BBTools). Barcode and primer sequences were removed from forward and reverse reads using cutadapt v3.0.[Ref.9] The dada2 v. 1.16.0, ref.[10] pipeline for paired-end reads was run with few modified parameters (filterAndTrim: truncLen=c(225,225); dada: pool=TRUE; assignTaxonomy: silva_nr_v138_train_set.fa.gz; addSpecies: silva_species_assignment_v138.fa.gz). Using R v. 4.0.3, output tables were merged, and all ASV taxonomically classified as "Chloroplast", "Archaea" or "Eukarya" as well as absolute singletons were removed from the dataset.

Alpha diversity was calculated using the subsamplingNGS.R function (https://github.com/chassenr/NGS/blob/master/Plotting/SubsampleNGS.R) with 100 subsamplings.

Phylogenetic analysis of Actinobacteriota.

Phylogenetic analysis of Actinobacteriota was done by using the software package ARB[11] based on the SILVA database SSU Ref NR, release 138.1. The tree was calculated by maximum-likelihood analysis using filters considering only positions (1389 positions) that are conserved in 50% of the sequences in a subset of 2518 high quality (pintail>50, seq_quality>70) nearly full-length 16S rRNA gene sequences (>1,350 bases). For all actinobacterial Svalbard $OTU_{0.98}$ ≥3 sequences, a representative sequence was inserted into the reconstructed tree by using parsimony criteria with global-local optimization, without allowing changes in the overall tree topology.

Automated imaging and counting

In sediments, counting fluorescently labeled microbial cells is challenging. Three major factors influence the quality of cell counts: signal to noise ratio, efficiency of sonication (dislodgement of cells from grains and separation of larger cell aggregates), and experience of the person who

counts. In particular, the latter should not be undervalued. In 2009, Morono and coworkers published the first automated system for cell enumeration in sediments.[12] Washing the sediments with hydrofluoric acid reduced non-biological background fluorescence. Further, SYBR Green I stained cells were distinguished from the background by a high ratio of green fluorescence (528/38 nm) versus red fluorescence (617/73 nm). The necessity of a strong acid for background reduction, however, does not allow this method to become standard. A low signal to background ratio in sediments compared to seawater required the use of narrow band pass optical filter sets to avoid the detection of unspecific signals. Number of layers for the imaged z-stack was increased to 13 (distance 0.4 μm) and the exposure time and minimal object size for cells were adjusted for each probe. Filter sections were imaged by automated microscopy using a motorized epifluorescence microscope (AxioImager.Z2m, Carl Zeiss, Jena, Germany) equipped with a 63x/1.4 Plan-Apochromat objective and controlled via the MPISYS software. Illumination was done using a Zeiss Colibri 7 LED source (385 nm for DAPI excitation and 469 nm for Alexa488 excitation) using specific narrow band pass optical filter sets for DAPI (splitter 375 nm; emission filter 448/20 nm) and Alexa488 (splitter 495 nm; emission filter 520/15 nm).

Greyscale images were recorded by a Zeiss AxioCam MRm with variable exposure times between 50 ms and 150 ms. For each field of view (FOV) a z-stack of 13 layers with a distance of 0.4 μm was recorded. For our sediment samples, up to 190 FOVs were imaged per filter section (average 139 FOVs +- STDEV 19, min 87 FOVs). A single extended depth of field image (EDF) was created from the individual images of a z-stack. The EDF compensates for the unevenness of the sample (e.g. aggregates or wrinkles on the filter piece) by taking multiple images corresponding to the different focal planes and subsequently creating a single in-focus image.

The images were quality proofed and analyzed using the Automated Cell Measuring and Enumeration tool 3.0 (ACMEtool) software (M. Zeder, www.technobiology.ch, [13]). In a first step, images that were out-of-focus or showed air bubbles were excluded from the analysis. At least 30 images per filter section (average 74 FOVs ± 27) passed this manual quality control routine. Subsequently, metadata file creation was done using the following parameters for object detection: for DAPI, kernel size 19, offset 11, remove regions smaller than 12 and larger than 5000; for CARD-FISH signals, kernel size 21, offset 11, remove regions smaller than 16 and larger than 5000. In a second step, cell identification by ACMEtool was done by filtering with the following parameters: for DAPI, $12 < \text{area} < 260$, a signal-to-background ratio (SBR) between 1.7 and 2.6, and a mean grey value p90 (MGVp90) between 39 and 81; for CARD-FISH signals, $20 < \text{area} < 750$, an SBR between 1.7 and 3.9, and a MGVp90 between 61 and 125.

About 15% of all FISH-stained cells did not show a corresponding DAPI due to quenching effects. Therefore, relative counts were obtained by enumeration of DAPI-stained cells on a different section of the same filter that passed the CARD-FISH procedure but without adding a probe. Total cell counts were determined on filter sections that were stained with DAPI only and did not pass the CARD-FISH procedure.

Table S1: Information on sampling sites in Svalbard Isfjorden, Arctic Ocean, and Helgoland Roads, North Sea. Helgoland chlorophyll *a*, temperature and salinity data provided by K. Wiltshire. NA, not analyzed

	Station	Lat [°N]	Long [°E]	Date [MM/DD/YYYY]	water depth [m]	Salinity [ppm]	Sediment temp. (Svalbard)/ Water temp. (Helgoland) [°C]	Chlorophyll *a* in seawater* [µg/l]	Chlorophyll *a* in sediment [µg/ml]	Phaeopigment in sediment [µg/ml]
Svalbard, Isfjorden	5	78.11	14.35	12/20/2017	8.84	NA	1.4	NA	0.3	0.5
				2/28/2018	5.5	NA	-0.6	NA	0.2	0.7
				5/1/2018	4.85	33	0.6	NA	1.0	0.5
				12/17/2018	4.01	35	0.6	0.4	0.3	0.4
				4/25/2019	4.84	35	2.2	8	0.4	0.4
				9/13/2019	6.14	32	4.5	1.8	1.8	2.8
	6	78.11	14.38	12/20/2017	6.55	35	1.4	NA	0.3	0.8
				2/28/2018	5.3	NA	-0.6	NA	0.3	0.5
				5/1/2018	5.78	34	0.6	NA	0.5	0.8
				12/17/2018	4.37	NA	0.5	0.4	0.4	0.6
				4/25/2019	4.84	35	1.9	6.3	0.5	0.4
				9/13/2019	6.25	NA	4.6	1.9	1.5	1.8
	7	78.10	14.38	12/20/2017	4.07	NA	1.5	NA	0.2	0.3
				2/28/2018	2.7	34	NA	NA	0.1	0.2
				5/1/2018	4.01	35	0.6	NA	1.5	0.4
				12/17/2018	2.95	33	0.5	0.4	0.2	0.2
				4/25/2019	2.48	35	2.0	6.8	0.2	0.2
				9/13/2019	2.7	33	4.5	2	0.4	0.7
	23	78.10	14.39	5/1/2018	4.62	33	0.6	NA	1.8	3.5
				12/17/2018	3.89	35	0.1	NA	0.2	0.4
				4/25/2019	3.9	35	2.1	NA	0.2	0.4
				9/13/2019	5.2	32.5	4.6	1.9	0.7	1.3
Helgoland	Helgoland Roads	54.18°N	7.90°E	1/23/2018	~3-8m	33	5.9	0.5	1.1	0.3
				3/7/2018		34	3.3	1.0	2.0	0.4
				4/17/2018		34	6.7	2.5	2.0	0.4
				5/4/2018		33	6.2	4.8♦	5.3	0.8
				5/15/2018		33	9.5	2.7	0.8	0.4
				7/12/2018		32	16.0	1.2	1.9	0.6
				9/5/2018		33	18.6	0.8♦	2.5	0.2
				11/7/2018		33	12.8	1.0♦	2.4	0.8
				12/5/2018		34	9.8	0.6♦	2.0	0.2
				1/18/2019		32	5.2	0.5♦	1.5	0.2
				3/8/2019		33	5.5	0.8♦	0.7	0.2
				3/27/2019		32	6.4	2.8♦	0.7	0.1
				4/5/2019		32	6.9	0.9♦	2.4	0.3
				4/16/2019		32	7.8	2.4	1.1	0.5
				5/2/2019		32	9.1	17.0	1.7	0.4
				5/16/2019		32	10.7	5.5	2.6	0.4
				7/10/2019		33	15.5	3.7♦	2.0	0.4
				9/23/2019		33	16.8	1.5$	1.4	0.5
				10/29/2019		33	13.7	0.9	2.3	1.4

*measured with a Cyclops-7 submersible sensor (Turner Designs, San Jose, USA)

$ measured the day after sediment sampling

♦ measured the day before sediment sampling

Table S2. Oligonucleotide probes used for CARD-FISH.

Probes*	Target	Sequence (5'-3')	FA conc.$	Target	Reference
EUB338 I	Bacteria	GCTGCCTCCCGTAGGAGT	35	16S rRNA	[14]
EUB338-II	Planctomycetota	GCAGCCACCCGTAGGTGT	35	16S rRNA	[15]
EUB338-III♦	Verrucomicrobiota	GCTGCCACCCGTAGGTGT	35	16S rRNA	[15]
NON338	Negative control	ACTCCTACGGGAGGCAGC	35	16S rRNA	[16]
GAM42a⊗	Gammaproteobacteria	GCCTTCCCACATCGTTT	35	23S rRNA	[17]
DELTA495a	Most Desulfobacterota, most Myxococcota, and other uncultured clades of former Deltaproteobacteria; most Gemmatimonadota	AGTTAGCCGGTGCTTCCT	35	16S rRNA	[18]
DELTA495b		AGTTAGCCGGCGCTTCCT	35	16S rRNA	[18]
DELTA495c		AATTAGCCGGTGCTTCCT	35	16S rRNA	[18]
DSS658	Desulfobacteraceae	TCCACTTCCCTCTCCCAT	50	16S rRNA	[19]
CF319a	Bacteroidota	TGGTCCGTGTCTCAGTAC	35	16S rRNA	[20]
PLA46	Planctomycetota except Phycisphaerae	GACTTGCATGCCTAATCC	30	16S rRNA	[21]
MIT1218	Microtrichales	AGCATGTTTGCAGCCCTG	25-30	16S rRNA	this study
c1_MIT1218	Competitor for MIT1218; diverse Phycisphaerae (320 sequ. ♣)	AGCACGTTTGCARCCCTG		16S rRNA	this study
c2_MIT1218	Competitor for MIT1218; Planctomycetota (145 sequ. ♣)	AGCACGTTTGCAGCCCMG		16S rRNA	this study
3H1_ MIT1218	Helper oligonucleotide for MIT1218	CKTTGTACCRGCCATTGT		16S rRNA	this study
5H2_ MIT1218	Helper oligonucleotide for MIT1218	GGCATAAGGGGCATGATG		16S rRNA	this study
ACM1218	Actinomarinales	AGCATGCGTGCAGCCCTG	25-30	16S rRNA	this study
c1_ACM1218	Competitor for ACM1218; class Actinobacteria (7800 sequ. ♣)	AGCATGCGTGAAGCCCTG		16S rRNA	this study
c2_ACM1218	Competitor for MIT1218; Actinobacteria (500 sequ. ♣)	AGCATGTGTGCAGCCCTG		16S rRNA	this study
5H1_ACM-MIT1218	helper oligonucleotide for ACM1218 and MIT1218	GAYATAAGGGGCATGATG		16S rRNA	this study
3H3_ACM1218	helper oligonucleotide for ACM1218	CGTTGTACHGGCCATTGT		16S rRNA	this study
3H3_ACM1218	helper oligonucleotide for ACM1218	CGTTGTACTTGCCATTGT		16S rRNA	this study
3H3_ACM1218	helper oligonucleotide for ACM1218	CGTTGTACCKGCCATTGT		16S rRNA	this study

*: for permeabilization, samples were treated with lysozyme (10 mg ml^{-1}, 0.05 M EDTA, 0.1 M Tris-HCl) at 37°C for 45 min. For hybridizations with probes Pla46, ACM1218 and MIT1218, samples were additionally treated with achromopeptidase (60 U ml^{-1}, 10 mM NaCl, 10 mM Tris-HCl, pH 8.0) at 37°C for 30 min.

$: formamide concentration in the hybridization buffer, hybridization at 46°C

♦: Probe EUB338-III was used at a molar ratio of 1:1 with competitor EUB338-II

⊗: Probe GAM42a was used at a molar ratio of 1:1 with the competitor Bet42a (GCCTTCCCACTTCGTTT)

♣: number of targeted sequences in arb database SSURef_NR99_132_SILVA_13_12_17_opt

Table S4. Bacterial diversity parameters from Svalbard sediments. Calculation based on 16S rRNA gene sequencing and subsequent amplicon sequence variant (ASV) analysis. Prior to analysis, chloroplast sequences, absolute singletons, and unspecifically amplified sequences from Archaea and Eukarya were removed. Mean spring was calculated from May 2018 and April 2019 data, mean winter was calculated from Dec2017 and Dec2018 data.

		sequences [no.]	ASV [observed]	ASV [subsampled*]	inv Simpson [subsampled*]	Shannon [subsampled*]
Sediment	st05_Dec2017	21278	3519	3063	109	6.5
	st06_Dec2017	42018	4127	2730	206	6.5
	st07_Dec2017	26729	3914	3052	101	6.4
	st05_Feb2018	20494	3726	3300	214	6.8
	st06_Feb2018	77749	6557	3521	286	7.0
	st07_Feb2018	24742	3861	3150	117	6.6
	st05_May2018	24053	4320	3531	186	6.9
	st06_May2018	22490	3917	3318	205	6.8
	st07_May2018	27962	4783	3647	254	6.9
	st23_May2018	21743	3821	3288	270	6.8
	st05_Dec2018	18257	3200	2992	108	6.4
	st06_Dec2018	16108	3324	3324	118	6.6
	st07_Dec2018	27402	4295	3273	129	6.6
	st23_Dec2018	26178	4253	3330	145	6.6
	st05_Apr2019	18765	3857	3565	186	6.8
	st06_Apr2019	17109	2953	2858	144	6.4
	st07_Apr2019	19789	2526	2278	71	5.8
	st23_Apr2019	30038	4378	3240	189	6.7
	st05_Sep2019	73024	6691	3670	328	7.1
	st06_Sep2019	19131	3811	3518	302	7.1
	st07_Sep2019	38301	4186	2734	58	6.0
	st09_Sep2019	31824	4583	3354	91	6.7
	**mean winter**			**3109**	**131**	**6.5**
	SD			218	36	0.1
	**mean spring**			**3215**	**188**	**6.6**
	SD			452	62	0.4
Seawater	st05_Feb2018	73612	3199	1641	26	5.2
	st07_Feb2018	53693	3259	1860	26	5.3
	st05_May2018	37520	1747	1200	7	3.4
	st07_May2018	45811	1734	1056	6	3.1
	st23_May2018	47036	2015	1291	9	3.7
	st05_Dec2018	87079	2909	1523	14	4.7
	st06_Dec2018	138979	3640	1594	16	4.9
	st07_Dec2018	100588	3023	1492	17	4.8
	st05_Apr2019	27957	1970	1494	14	4.1
	st06_Apr2019	23727	1910	1583	20	4.5
	st07_Apr2019	24713	1817	1488	16	4.3
	st23_Apr2019	30988	2048	1483	15	4.2
	st05_Sep2019	71405	2549	1318	18	4.2
	st06_Sep2019	129408	2703	1193	9	3.9
	st07_Sep2019	134439	2813	1199	8	3.9
	**mean winter**			**1536**	**16**	**4.8**
	SD			52	2	0.1
	**mean spring**			**1371**	**12**	**3.9**
	SD			192	5	0.5

*Subsampling was performed using 16108 sequences per sample.

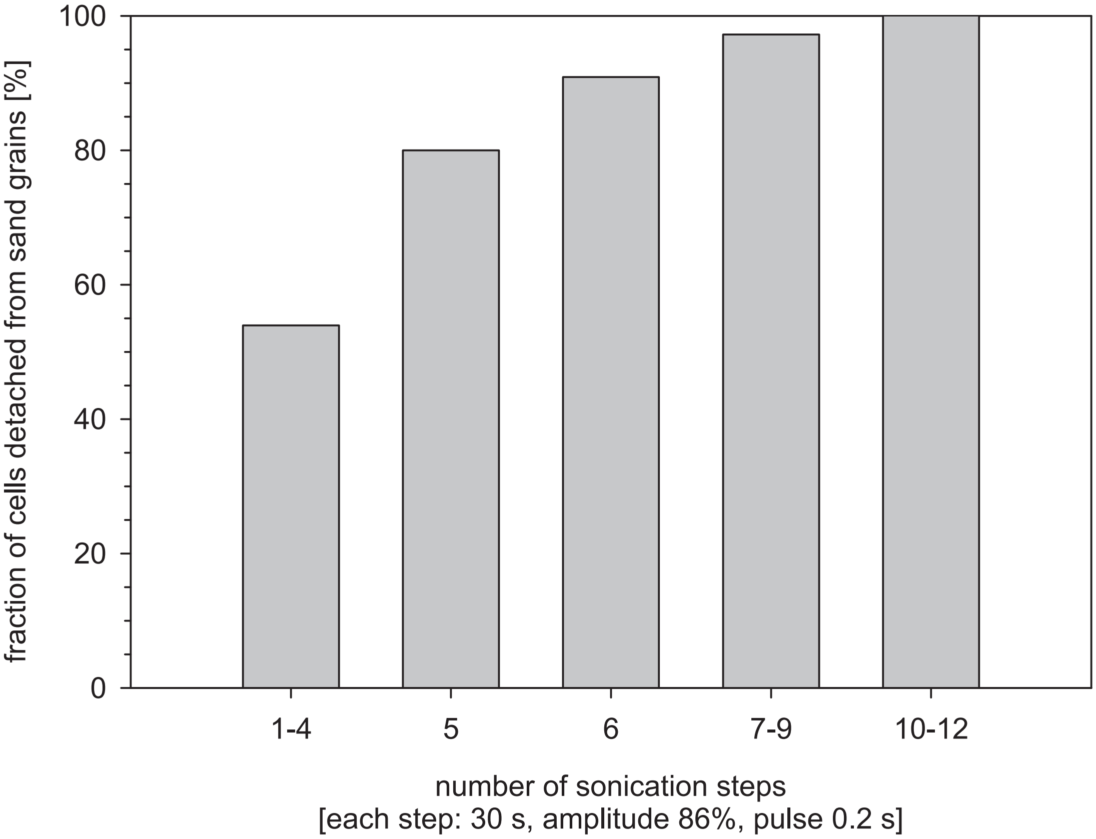

Supplementary Figure S1

Efficiency of sonication treatment on the detachment of microbial cells from sand grains. Formaldehyde-fixed sediments from Svalbard, station 13 (May 2018, 78.11°N, 14.33° E), were treated several times by sonication using a type MS2.5 probe (Sonopuls mini20, Bandelin) for 30 s at an amplitude of 86% and pulse of 0.2 s. After each step, the supernatant was removed and collected in a new tube. An appropriate volume of the supernatant of each step was filtered onto 0.2 µm pore size polycarbonate filters. Filter sections were mounted on glass slides using a mixture of glycerin–phosphate-buffered saline (PBS) mounting solutions (CitiFluorAF1 [CitiFluor Ltd., London, United Kingdom] and Vectashield [Vector Laboratories, Burlingame, CA, USA]) containing the nucleic acid dye DAPI (4',6-diamidino-2-phenylindole; Sigma-Aldrich, Steinheim, Germany) at a final concentration of 1 µg ml^{-1}. After sonication step 6, 91% of total cells were recovered as determined by epifluorescence microscopy and manual cell counting. From sonication steps 7 to 12, 9% additional cells could be retrieved, however, cells were often damaged and disruption into several parts could not be excluded.

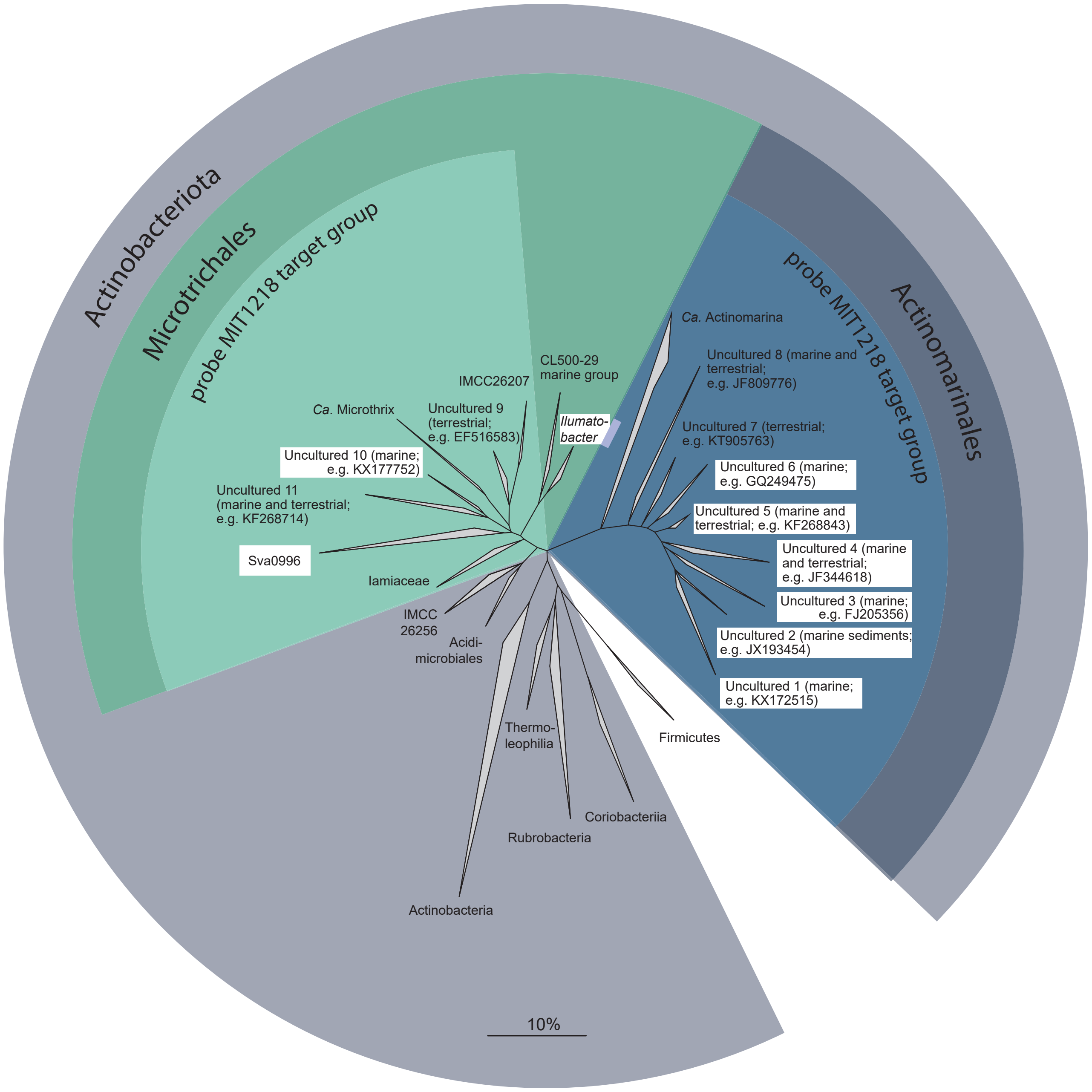

Supplementary Figure S2. Phylogenetic tree showing the phylum Actinobacteriota.

Of 1703 Actinobacteriota Svalbard $OTU_{0.98}$, 937 $OTU_{0.98}$ affiliated with Actinomarinales (55% of Actinobacteriota sequences) and 710 OTU0.98 with Microtrichales (37%). Of these, 39% and 41% of Actinomarinales sequences were affiliated with clades "Uncultured 1" and "Uncultured 6"; of Microtrichales sequences, 21%, 33% and 46% were affiliated with clades "Sva0996", "Uncultured 10" and *Ilumatobacter* spp., respectively. Probe specificity of designed probes for Actinomarinales (probe ACM1218) and Microtrichales (MIT1218) is indicated. Group coverage of probe ACM1218 is 92% and of probe MIT1218 is 57%. *Ilumatobacter*, IMCC26207, and CL500-29 marine group are not targeted by MIT1218, thus explaining its low coverage. Bar = 10% estimated sequence divergence

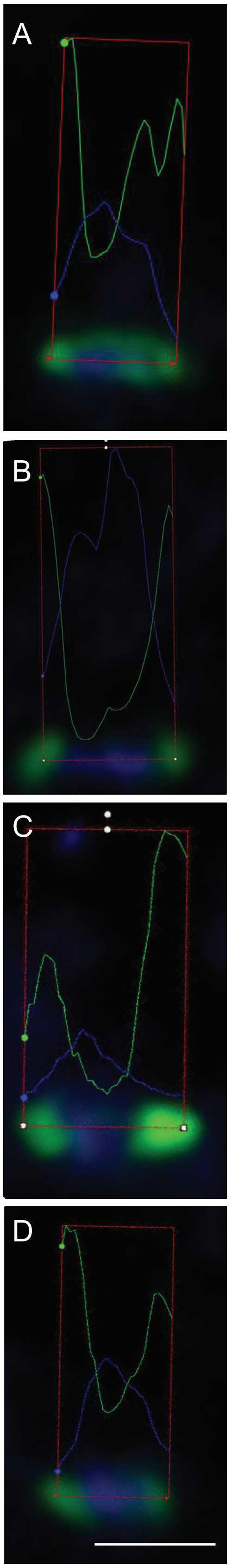

Supplementary Figure S3

Identification of dividing Actinomarinales cells using laser scanning micrographs. The fraction of dividing cells was determined based on line profiles of DAPI (blue) and FISH signal fluorescence (green, probe ACM1218). Dividing cells were defined by two criteria: i) two DAPI maxima are visible in a single cell and ii) one or no FISH signal maximum is present in the cell center between the two DAPI maxima. If two FISH signal maxima were detected in the cell center, cell division is categorized as completed. According to these criteria, panels A, B show dividing cells, and panels C, D show non-dividing cells

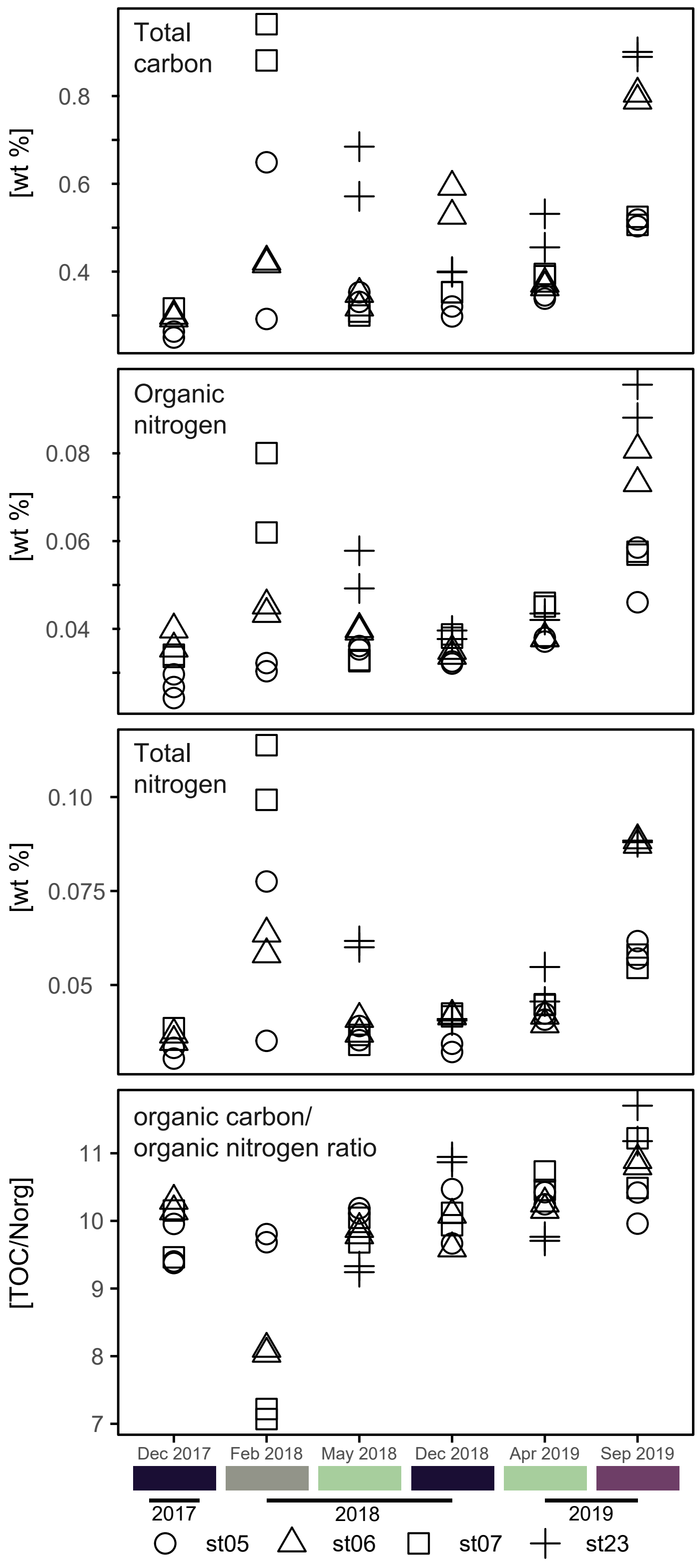

Supplementary Figure S4

Total carbon, organic nitrogen, total nitrogen, and C/N (organic carbon/organic nitrogen) ratio in Svalbard sediments. Seasons are color-coded (winter: black, twilight: grey; spring: green; summer/autumn: aubergine-colored).

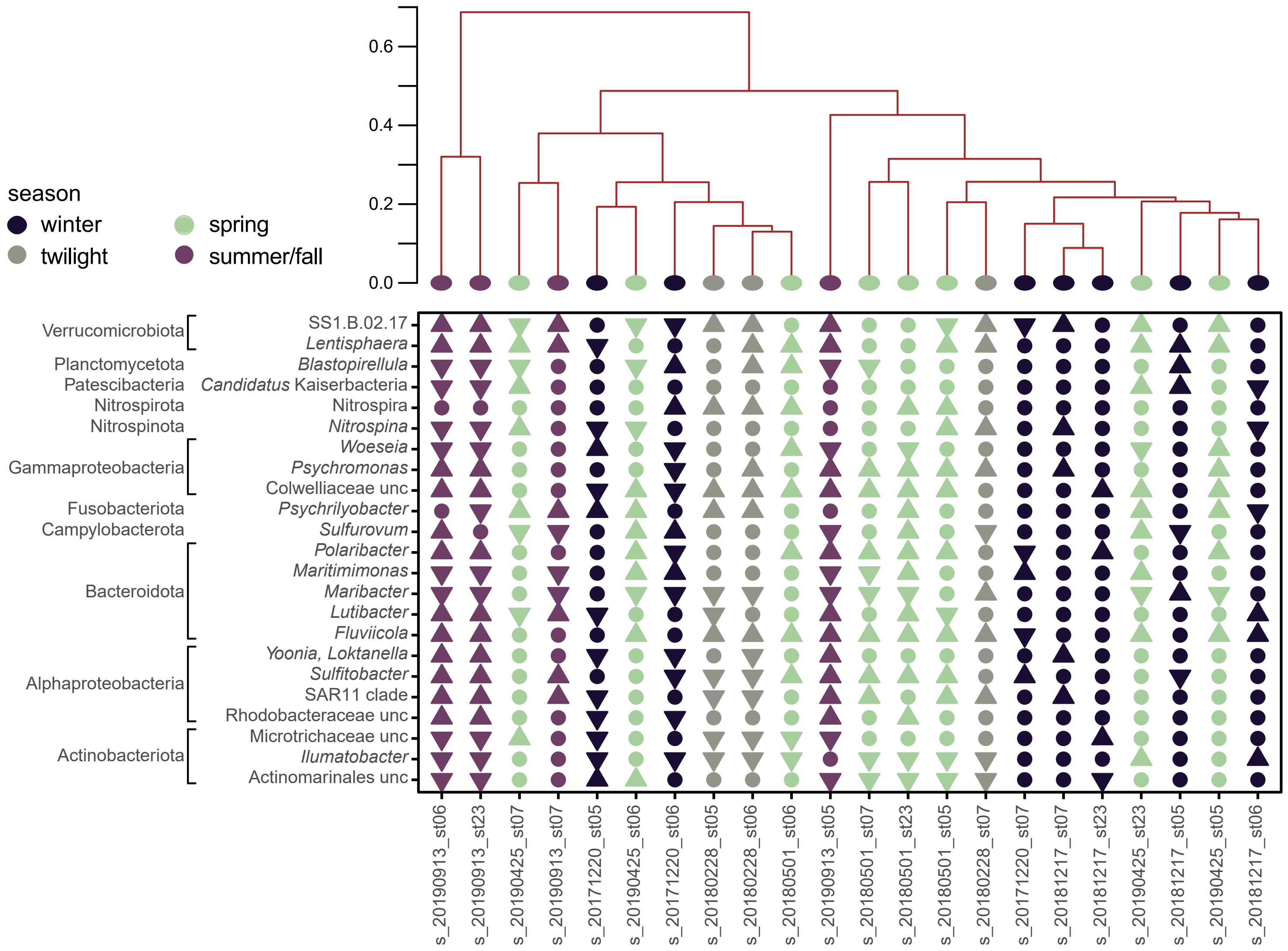

Supplementary Figure S5

Hierarchical clustering analysis of samples, based on Bray Curtis dissimilarity, and changes of the bacterial community composition in Svalbard sediments. Changes based on 16S rRNA gene frequencies are indicated as significant decrease (arrow down) or increase (arrow up) when they differed by more than 1% to the mean ± SD as calculated for each taxon of the seven winter samples. Changes of relative abundance with ±1% to SD were considered as no change (o).

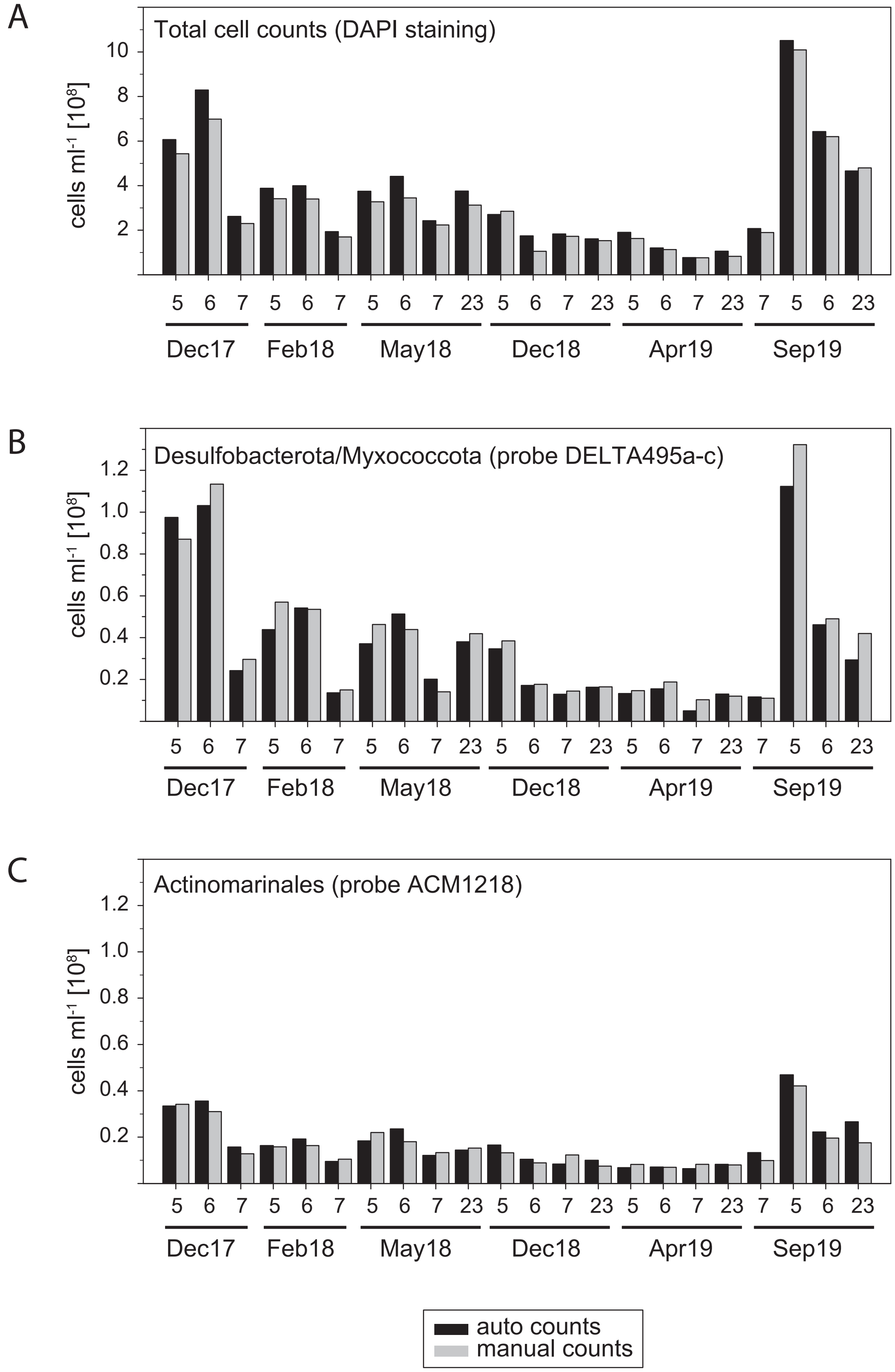

Supplementary Figure S6

Evaluation of automated cell counting in Svalbard sediments. Filter sections stained with DAPI (panel A) or hybridized with HRP-labelled probes DELTA495a-c (panel B) or ACM1218 (panel C) were imaged by automated microscopy and analyzed using the ACMEtool software for cell identification and counting (black bars). On average, 74 fields of view were analyzed. Afterwards, 30 fields of view were manually counted on the same filter sections (grey bars). A comparison of automated counts versus manual counts for 22 samples showed that manual counts constituted 89±10%, 112±26% and 98±22% of automated counts for total DAPI cell counts, probe DELTA495a-c, and probe ACM1218, respectively.

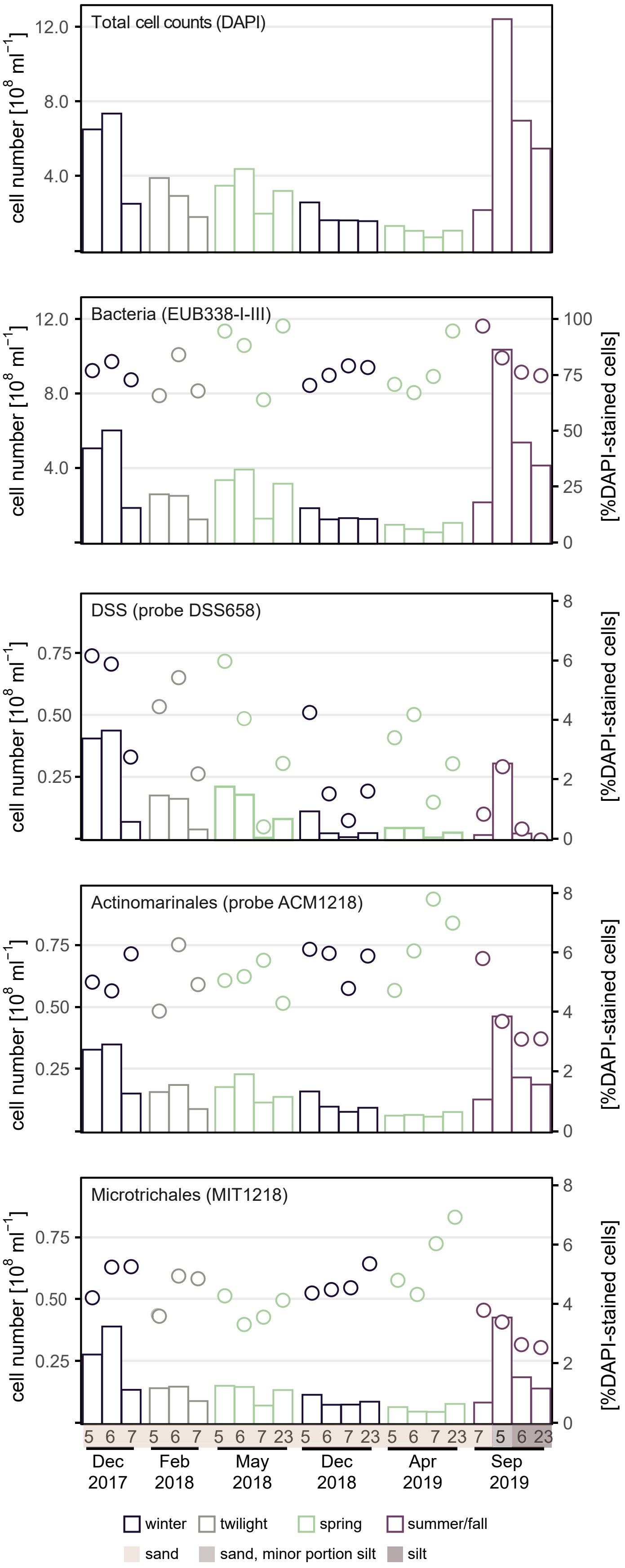

Supplementary Figure S7

Total cell counts and in situ abundance of selected bacterial taxa in Svalbard sediments as determined by DAPI staining and CARD FISH. Cell numbers were determined by automated microscopy and subsequent image analysis. Bars give absolute cells numbers, circles give the fraction of DAPI-stained cells. Color code reflects the season in which the samples were taken. Note the different scale on y-axes.

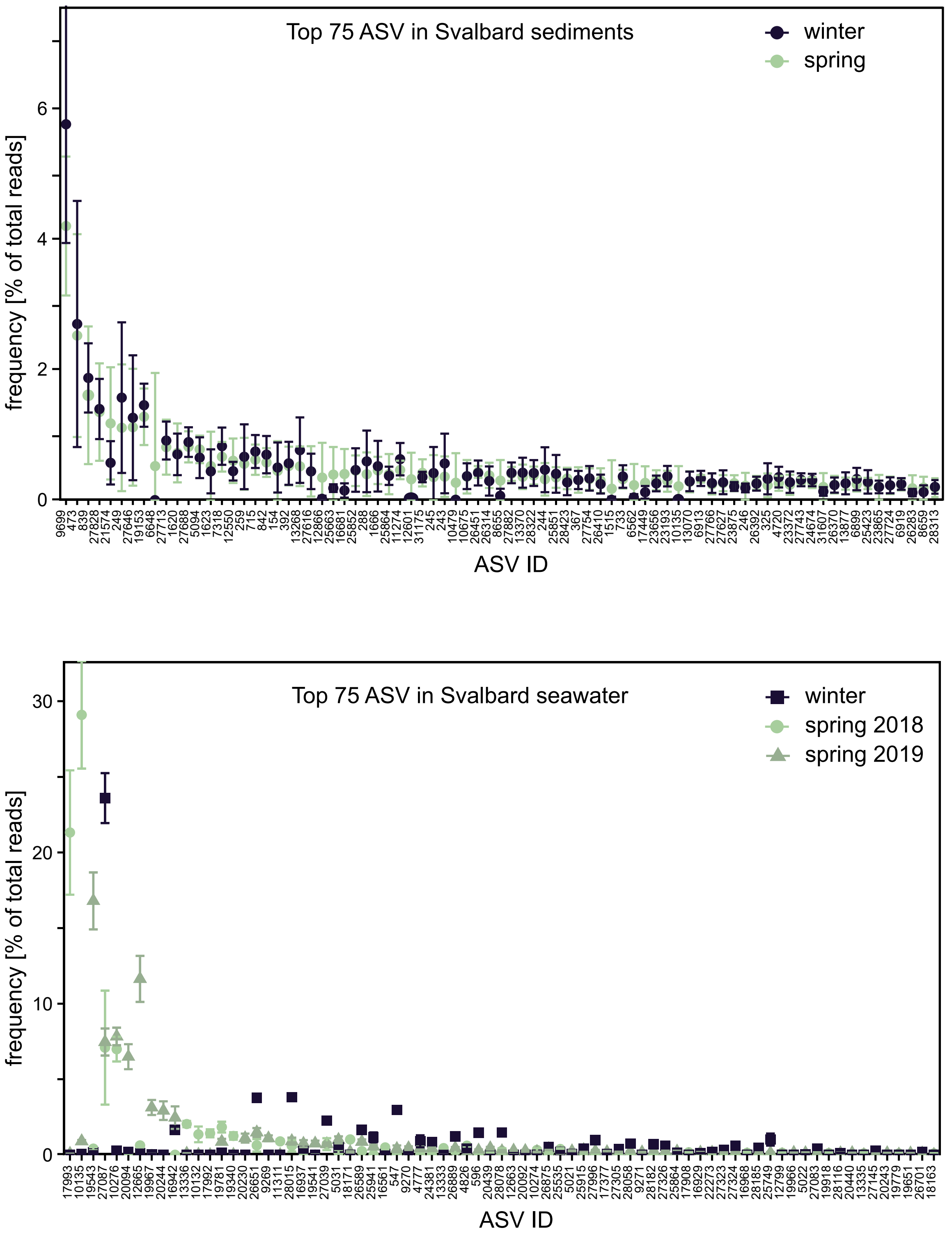

Supplementary Figure S8

Mean frequencies of most abundant amplicon sequence variant (ASV) from Svalbard seawater and sediment samples based on partial 16S rRNA gene sequencing. ASV frequencies in winter and spring are shown in comparison. In water samples, frequencies of several ASV differ significantly between winter and spring while those in sediments only changed within standard deviation.

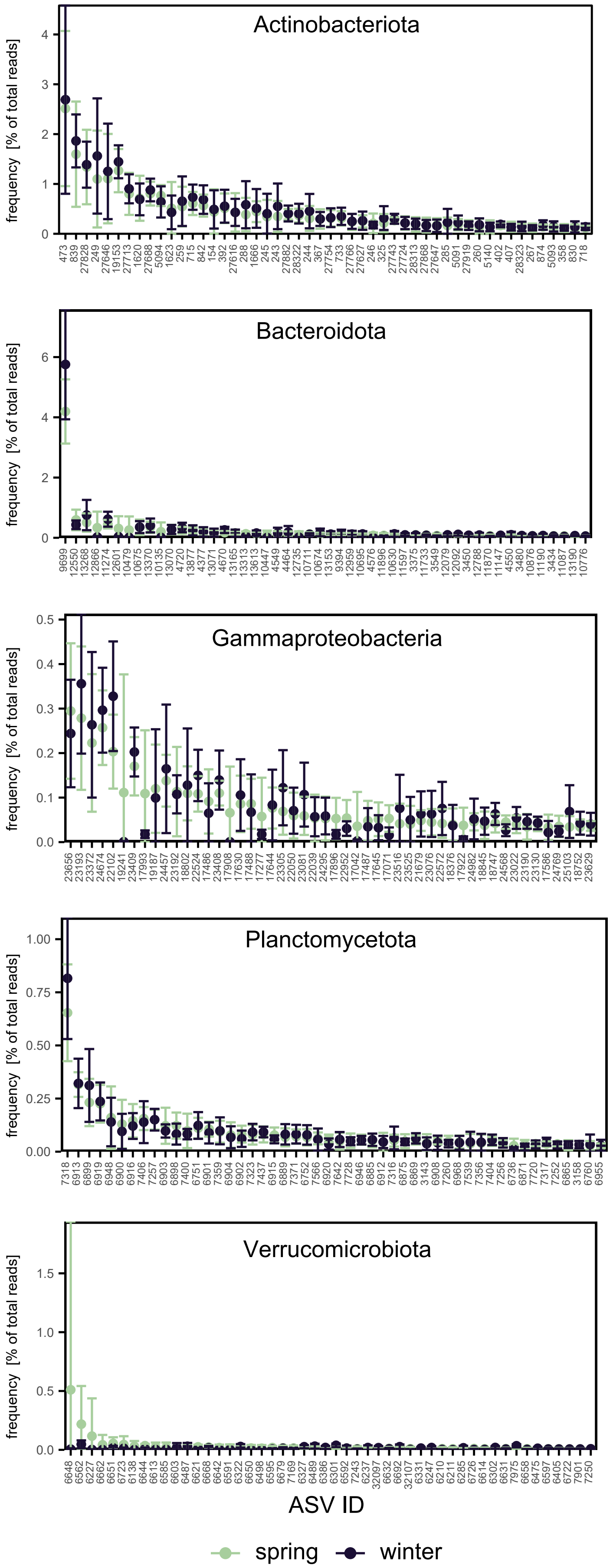

Supplementary Figure S9

Mean frequencies of top 50 most abundant amplicon sequence variant (ASV) from Svalbard sediment samples based on partial 16S rRNA gene sequencing. ASV frequencies in winter and spring are shown in comparison. Frequencies in ASV of major taxa did not differ significantly between winter and spring. Note the different scale of y-axes for the plots.

References

1. McGregor H.V., Dupont L.M., Stuut J.-B.W., Kuhlmann H. Particle size analysis and end-member modelling of sediment core GeoB6008-2. In supplement to: McGregor, HV et al. (2009): Vegetation change, goats, and religion: a 2000-year history of land use in southern Morocco. Quaternary Science Reviews, 28(15-16), 1434-1448, https://doi.org/10.1016/j.quascirev.2009.02.012: PANGAEA; 2009.
2. Herlemann D.P.R. et al. Transitions in bacterial communities along the 2000 km salinity gradient of the Baltic Sea. *ISME J.* **5**, 1571-1579 (2011).
3. Andrews S. FASTQC. A quality control tool for high throughput sequence data. 2010.
4. Bushnell B., Rood J., Singer E. BBMerge - Accurate paired shotgun read merging via overlap. *PLoS One* **12**, e0185056 (2017).
5. Schloss P.D. et al. Introducing mothur: Open-source, platform-independent, community-supported software for describing and comparing microbial communities. *Appl. Environ. Microbiol.* **75**, 7537–7541 (2009).
6. Quast C. et al. The SILVA ribosomal RNA gene database project: improved data processing and web-based tools. *Nucleic Acids Res.* **41**, D590-D596 (2013).
7. Team R.C. R: A language and environment for statistical computing. Vienna, Austria: R Foundation for Statistical Computing, URL https://www.r-project.org/; 2019.
8. Wickham H. tidyverse: Easily Install and Load the 'Tidyverse'. 2017.
9. Martin M. Cutadapt removes adapter sequences from high-throughput sequencing reads. *EMBnet J.* **17**, 10-12 (2011).
10. Callahan B.J., McMurdie P.J., Holmes S.P. Exact sequence variants should replace operational taxonomic units in marker-gene data analysis. *ISME J.* **11**, 2639-2643 (2017).

11. Ludwig W. et al. ARB: a software environment for sequence data. *Nucleic Acids Res.* **32**, 1363-1371 (2004).

12. Morono Y., Terada T., Masui N., Inagaki F. Discriminative detection and enumeration of microbial life in marine subsurface sediments. *ISME J.* **3**, 503-511 (2009).

13. Bennke C.M. et al. Modification of a high-throughput automatic microbial cell enumeration system for shipboard analyses. *Appl. Environ. Microbiol.* **82**, 3289-3296 (2016).

14. Amann R.I. et al. Combination of 16S rRNA-targeted oligonucleotide probes with flow cytometry for analyzing mixed microbial populations. *Appl. Environ. Microbiol.* **56**, 1919-1925 (1990).

15. Daims H., Brühl A., Amann R., Schleifer K.-H., Wagner M. The domain-specific probe EUB338 is insufficient for the detection of all bacteria: development and evaluation of a more comprehensive probe set. *Syst. Appl. Microbiol.* **22**, 434-444 (1999).

16. Wallner G., Amann R., Beisker W. Optimizing fluorescent in situ hybridization with rRNA-targeted oligonucleotide probes for flow cytometric identification of microorganisms. *Cytometry* **14**, 136-143 (1993).

17. Manz W., Amann R., Ludwig W., Wagner M., Schleifer K.-H. Phylogenetic oligodeoxynucleotide probes for the major subclasses of *Proteobacteria*: problems and solutions. *Syst. Appl. Microbiol.* **15**, 593-600 (1992).

18. Lücker S. et al. Improved 16S rRNA-targeted probe set for analysis of sulfate-reducing bacteria by fluorescence in situ hybridization. *J. Microbiol. Methods* **69**, 523-528 (2007).

19. Manz W., Eisenbrecher M., Neu T.R., Szewzyk U. Abundance and spatial organization of gram-negative sulfate-reducing bacteria in activated sludge investigated by in situ probing with specific 16S rRNA targeted oligonucleotides. *FEMS Microbiol. Ecol.* **25**, 43-61 (1998).

20. Manz W., Amann R., Ludwig W., Vancanneyt M., Schleifer K.-H. Application of a suite of 16S rRNA-specific oligonucleotide probes designed to investigate bacteria of the phylum cytophaga-flavobacter-bacteroides in the natural environment. *Microbiology* **142**, 1097-1106 (1996).

21. Neef A., Amann R., Schlesner H., Schleifer K.H. Monitoring a widespread bacterial group: in situ detection of planctomycetes with 16S rRNA-targeted probes. *Microbiology (Reading)* **144**, 3257-3266 (1998).
